# Using an external electric field to tune active layer morphology enabling high-efficiency organic solar cells via ambient blade coating

**DOI:** 10.1126/sciadv.ado5460

**Published:** 2024-06-28

**Authors:** Fengzhe Cui, Jiawei Qiao, Yujie Xu, Zhen Fu, Ruohua Gui, Chen Zhang, Rongkun Zhou, Long Ye, Xiaoyan Du, Feng Chen, Xiaotao Hao, He Yan, Hang Yin

**Affiliations:** ^1^ School of Physics, State Key Laboratory of Crystal Materials, Shandong University, 250100 Shandong, Jinan, China.; ^2^ Department·of Computing, The Hong Kong Polytechnic University, 11 Yuk Choi Road, Hung Hom, Kowloon, Hong Kong 999077, China.; ^3^ School of Materials Science and Engineering, Tianjin Key Laboratory of Molecular Optoelectronic Sciences, Tianjin University, 300072 Tianjin, China.; ^4^ Department of Chemistry and Hong Kong Branch of Chinese National Engineering Research Center for Tissue Restoration and Reconstruction, Hong Kong University of Science and Technology, Clear Water Bay, Kowloon, Hong Kong 999077, China.

## Abstract

The nanoscale morphology of the photoactive layer notably impacts the performance of organic solar cells (OSCs). Conventional methods to tune the morphology are typically chemical approaches that adjust the properties (such as solubility and miscibility) of the active components including donor, acceptor, and/or additive. Here, we demonstrate a completely different approach by applying an external electric field (EEF) on the active layer during the wet coating. The EEF-coating method is perfectly compatible with an ambient blade coating using environmentally friendly solvents, which are essential requirements for industrial production of OSCs. A record 18.6% efficiency is achieved using the EEF coating, which is the best value for open-air, blade-coated OSCs to date. Our findings suggest broad material applicability and attribute-enhanced performance to EEF-induced fiber formation and long-range ordering of microstructures of acceptor domains. This technique offers an effective method for producing high-performance OSCs, especially suited for industry OSC production based on open-air printing.

## Teaser

External electric field manipulates the nanoscale morphology of organic thin films through interactions with molecular dipoles.

## INTRODUCTION

Organic solar cells (OSCs) are one of the leading candidates for next-generation solar technologies, owing to their attractive features such as lightweight, flexibility, and low-cost fabrication (*1*–*5*). The morphology of the photoactive layer is one of the most important factors determining the photovoltaic performances of OSCs (*6*–*10*). The nanostructure of the active layer in OSCs is multidimensional, mainly involving aspects such as domain size, domain purity, donor-acceptor miscibility, material crystallinity, and molecular orientations (*11*–*14*). Among various factors, achieving a bicontinuous interpenetrating fibrous network is particularly important for the morphology of bulk-heterojunction (BHJ) films (*3*, *8*, *15*). Such fibrous nanostructure has been achieved in some previous studies, in which the donor polymers can pre-aggregate into nanometer-size fibers that provide a highway of hole transport (*16*–*18*). On the other hand, small molecular acceptors (SMAs) are much shorter in length than polymers and do not have the aggregation property inherent to typical donor polymers. As a result, SMAs might not be able to form fibrous structures as easily as donor polymers (*19*). Therefore, it is important to develop methodologies that facilitate the formation of fibrous nanostructures within SMA domains to establish a bicontinuous fibrous network for both donors and acceptors. With this background, researchers in the OSC community are devoted to developing systematic methods to optimize BHJ morphology and enhance the performance of OSCs.

Most reported methods to optimize the BHJ morphology are chemical methods that focus on molecular designs, solvent and additive selections, and compositional adjustments (*20*, *21*). These chemical methods are shown to be highly effective in enhancing OSC performances. Besides chemical methodologies, there are also some other attempts of using physical methods—such as magnetic fields, electric fields, and alternating electromagnetic fields—to control the morphology of the active layer (*22*, *23*). However, the experimental attempts to implement such physical methods have not been exceptionally successful and the best efficiency achieved was only about 11% using these methods (*24*, *25*). The reasons for these unsuccessful attempts were not investigated in detail, but could be due to some of the following factors. For example, previous works of using electric or magnetic fields were applied on the active layers that are already mostly dried. In such conditions, it would be difficult to significantly change the nanostructure of the solid films as the molecules have limited mobility in the solid state (*22*, *26*). In another study, the authors had to introduce inorganic magnetic particles into the BHJ blends, possibly leading to negative effects on the morphology and charge separation process within the active layers (*25*). Although these initial attempts of using physical methodologies were not highly successful, it is still worthwhile to further investigate physical approaches, as they may offer morphological effects that cannot be achieved using conventional chemical methods.

Here, we demonstrate a unique nonchemical approach that employs an external electric field (EEF) to tune the morphology of photoactive layers in the wet coating process (not after the film is already dried). This method is highly effective in improving the BHJ morphology and device performance over a number of SMA-based OSC systems, showing its broad applicability. Morphological study reveals that the EEF treatment can introduce the formation of fibrous structure in the SMA domains with improved charge carrier mobility. The EEF method would not be effective if it is applied to photoactive films that have already dried; our method can only be effective when the EEF is applied to wet solutions during the coating process of active layers. It is reasonable to expect that EEF could influence the alignment and orientation of SMAs in solution, given their strong dipole moments, making them readily responsive to EEF in the solution rather than in the solid state. To characterize the fibrous nanostructure formed by SMAs, we perform photo-induced force microscopy (PiFM) measurement that can selectively map the nanostructure of SMA domains. The PiFM images confirm that EEF can contribute directly to the formation of fibrous nanostructures in the SMA domains. Our EEF-coating method is compatible with the industrial open-air printing process using eco-friendly solvent, achieving an efficiency of 18.6%, the highest performance reported to date for OSCs fabricated under these industrial relevant conditions. Our work not only offers a physical method to tune the active layer morphology with broad applicability but also is compatible with industrial processes, thereby accelerating the industrial application of OSC materials and technologies.

## RESULTS

### EEF coating and device performances

**Fig. 1.**
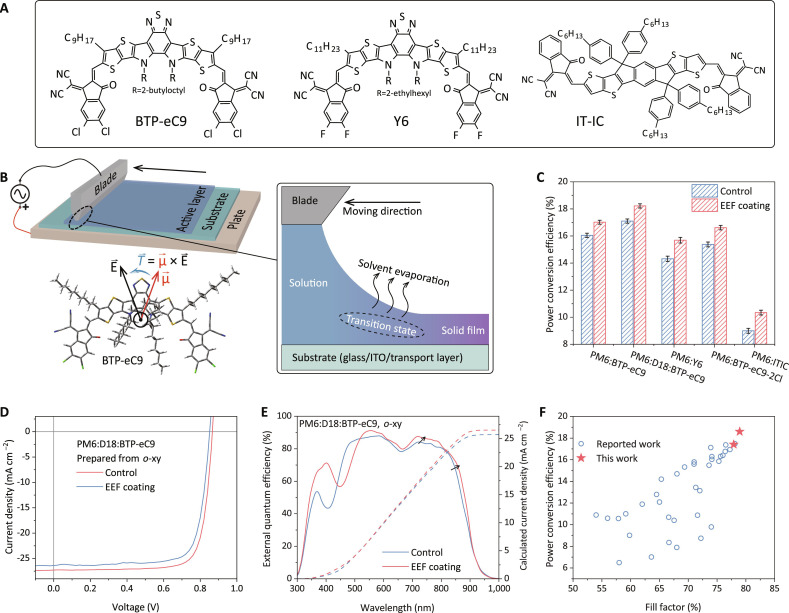
Experimental equipment schematic and photovoltaic properties. (**A**) Chemical structures of NFA materials used in this work. (**B**) Schematic diagram of the experimental equipment, partial enlargement, and the torque of an electric field on a molecule. (**C**) Column charts with error bars (*n* = 50) of PCE distributions of OSC devices using a variety of NFAs. (**D**) *J*-*V* characteristics of PM6:D18:BTP-eC9 devices under simulated air mass 1.5 global (AM 1.5 G) illumination at 100 mW cm^−2^. (**E**) Corresponding EQE spectra (solid lines) and integrated *J*_sc_s (dashed lines) of the PM6:D18:BTP-eC9 devices. (**F**) Statistical plots of PCE versus FF for reports of OSCs with active layers prepared in air, with corresponding data points and references listed in table S2.

The experimental setup of our EEF guided blade coating and chemical structures of non-fullerene acceptors (NFAs) is shown in Fig. 1 (A to B) (with additional details of EEF-coating setup provided in Materials and Methods). On the basis of this EEF-coating setup, we fabricated OSCs using a variety of NFA photoactive materials to evaluate the effect of EEF on photovoltaic performance (Fig. 1C). The current density-voltage (*J*-*V*) curves under AM 1.5 G illumination and external quantum efficiency (EQE) characteristics are summarized in Table 1 and Fig. 1 (D and E), with figs. S7 to S10 providing additional data. With the EEF coating, all device parameters—short circuit current density (*J*_SC_), open circuit voltage (*V*_OC_), and fill factor (FF)—across all studied material systems significantly enhanced, resulting in at least 1% boost in the power conversion efficiency (PCE). In particular, the EEF-coated PM6:D18:BTP-eC9 (*o*-xy) device achieved a maximum PCE of 18.6%, featuring a *V*_OC_ of 0.866 V, a *J*_SC_ of 26.3 mA cm^−2^, and an FF of 78.9%, representing the highest reported PCE for OSCs prepared in an open-air environment (Fig. 1F). Our method is not only applicable to different material systems, but also compatible with industry-relevant processing conditions. The EEF coating also enabled enhanced performance OSCs with thick active layers and large device areas, attributes that hold significant promise for industrial manufacturing. Meanwhile, EEF-coated devices have demonstrated improved operational stability (fig. S11 and text S4), which can be used to evaluate the benefits of commercialization. In addition to *o*-xy mentioned above, chloroform (CF) with higher polarity has also been explored in detail using solvent as a reference and showed performance enhancement of EEF-coated photovoltaic devices (figs. S12 to S16, table S3, and text S5).

**Table 1.** Summary of photovoltaic parameters of the PM6:BTP-eC9 devices prepared in different methods.

Samples		*J*_sc_ (mA cm^−2^)	*J*_cal._ (mA cm^−2^) *	*V*_oc_ (V)	FF (%)	PCE (%)†
PM6:BTP-eC9 (*o*-xy)	Control	25.4	24.8	0.842	76.7	16.4 (16.0 ± 0.2)
	EEF coating	26.3	26.1	0.851	77.9	17.4 (17.0 ± 0.1)
PM6:BTP-eC9 (*o*-xy, thermal annealed)	Control	25.8	25.1	0.847	77.2	16.9 (16.5 ± 0.2)
	EEF coating	26.3	26.1	0.850	77.8	17.4 (17.0 ± 0.2)
PM6:D18:BTP-eC9 (*o*-xy)	Control	26.5	25.7	0.851	77.5	17.5 (17.1 ± 0.2)
	EEF coating	27.2	26.5	0.866	78.9	18.6 (18.2 ± 0.2)
PM6:BTP-eC9 (*o*-xy, ~300 nm)	Control	26.2	25.7	0.818	70.1	15.0 (14.6 ± 0.2)
	EEF coating	27.2	26.8	0.830	71.4	16.1 (15.7 ± 0.2)
PM6:BTP-eC9 (*o*-xy, 1 cm^2^)	Control	21.8	21.4	0.849	75.4	14.0 (13.6 ± 0.2)
	EEF coating	23.0	22.2	0.863	77.1	15.3 (14.9 ± 0.2)
PM6:Y6 (*o*-xy)	Control	25.7	–	0.826	69.8	14.8(14.3 ± 0.3)
	EEF coating	26.5	–	0.844	71.8	16.1(15.7 ± 0.2)
PM6:BTP-eC9-2Cl (*o*-xy)	Control	24.4	–	0.874	74.0	15.8(15.3 ± 0.3)
	EEF coating	25.3	–	0.886	75.9	17.0(16.6 ± 0.3)
PM6:ITIC (*o*-xy)	Control	14.7	–	0.960	66.6	9.4(8.9 ± 0.2)
	EEF coating	16.1	–	0.974	68.3	10.7(10.3 ± 0.2)

*Integrated current densities from EQE curves.

†Average values with SD were obtained from 50 or 30 devices (*n* = 30 for thick film and 1 cm^2^ devices, and *n* = 50 for the remaining conditions).

**Fig. 2.**
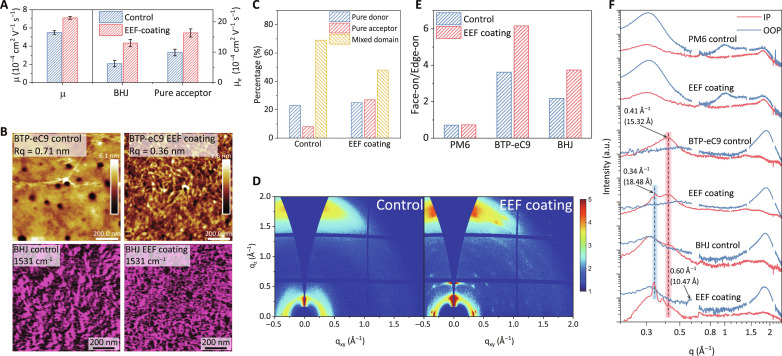
Surface morphology and molecular packing of thin films. (**A**) Carrier mobilities of the control and EEF-coated blended and pure acceptor (*o*-xy) devices acquired from space charge–limited current (SCLC) and photo-induced carrier extraction by linearly increasing voltage (photo-CELIV) measurements. (**B**) AFM height maps of PM6 (*o*-xy), BTP-eC9 (*o*-xy), and PM6:BTP-eC9 (*o*-xy) films. PiFM images at a wave number of 1531 cm^−1^ (representing BTP-eC9 N-H stretching). (**C**) The percentages of pure domains and mixed domains obtained by segmenting different areas and performing corresponding calculations in integrated PiFM images. (**D**) The 2D GIWAXS patterns of PM6:BTP-eC9 (*o*-xy) blends. q_xy_, scattering vector in the IP direction; q_z_, scattering vector in the out-of-plane (OOP) direction. (**E**) The face-on/edge-on ratio obtained from pole figures extracted from the (010) diffractions for corresponding films. (**F**) Line cut profiles of 2D GIWAXS data along the IP and OOP directions. The gaps on the lines caused by the gaps on the detector.

The charge carrier mobilities of control and EEF-coated devices were evaluated to understand the reasons for the enhanced photovoltaic performances. First, using a conventional space charge–limited current (SCLC) method, the control device obtained an electron mobility (μ_e_) of 6.2 × 10^−4^ cm^2^ V^−1^ s^−1^, while the EEF-coated device obtained a μ_e_ of 1.3 × 10^−3^ cm^2^ V^−1^ s^−1^ (Fig. 2A), which is one of the highest values reported for NFA-based OSCs. The photo-induced carrier extraction by linearly increasing voltage (photo-CELIV) further shows that the EEF-coated blends have a higher capability in carrier transport (fig. S17 and table S4). The carrier mobilities (μ) have been enhanced from 5.5 × 10^−4^ cm^2^ V^−1^ s^−1^ to 7.1 × 10^−4^ cm^2^ V^−1^ s^−1^ in the EEF-regulated devices (Fig. 2A). For control devices, the lower electron mobility set the fundamental limitations for charge transport, hence limiting the device performance. The EEF leads to higher mobility in EEF-coated devices, thus reducing the likelihood of bimolecular and trap-assisted recombination and enhance the performances of OSCs.

### Morphology details of the NFA fibrous network

To understand the enhanced photovoltaic performances and electron mobilities, we further investigated the nanomorphology of the EEF-coated films. First, we used atomic force microscope (AFM) analysis to investigate the surface topography (figs. S18 to S20) of the *o*-xy processed neat films with or without EEF coating. As shown in Fig. 2B, the neat NFA films with EEF coating show distinct fibrous nanostructures that are not visible for the samples without EEF coating. Then, we investigate the nanostructures of the blend films and find that both the control and EEF-coated cases exhibit fibrous nanostructures. This is likely due to the fact that the donor polymer can form fibrous nanostructure in blend films. Apparently, AFM images do not distinguish between donor and acceptor and therefore cannot selectively map the fibrous nanostructure formed by the NFAs. To selectively map the nanostructure of NFAs, we resort to the PiFM technique, which was demonstrated to selectively image either donor or acceptor nanostructures through the analyses of stretching of feature bonds (Fig. 2B and figs. S21 to S24). On the basis of the PiFM results, we find that the acceptor domains in EEF-coated BHJ demonstrate a clear fibrous structure with a diameter of about 20 nm (fig. S25), which is similar to the fiber size of those revealed by the AFM image of the neat film. In addition, overlaying the PiFM images of PM6 and BTP-eC9 (figs. S26 to S28 and text S6) yields a bicontinuous fibrous network of BHJ films, wherein the donor and acceptor domains closely contacted each other. With the combination of AFM images of neat films and PiFM images of blend films, we can draw the conclusion that the EEF indeed induces the formation of fibrous structure in acceptor domains.

Besides this information, the PiFM images also provide other insights regarding the reduced mixed domain fraction and elevated pure acceptor domain fraction for the EEF-coated films (Fig. 2C). We have also verified the fibrous structure of acceptor domains in heterojunctions using transmission electron microscopy (TEM) studies. As shown by TEM investigation (fig. S29), a fibrous network structure can be seen in all blend films. The fibrous texture in primitive PM6:BTP-eC9 blends is roughly defined with a larger acceptor domain and more sparse fibrous distribution, while the electron-rich regions (acceptor domains) in the EEF-coated blends formed a more continuous fiber-like structure, as we observed in PiFM (*27*).

To delve into the NFA fibrous nanostructure, we utilized grazing-incidence wide-angle and small-angle x-ray scattering (GIWAXS/GISAXS) characterizations (Fig. 2D, figs. S24 to S26, and tables S4 to S8). Integration along the polar angle direction suggests that EEF significantly enhances the face-on microcrystal ratio of receptor molecules in neat and BHJ films (Fig. 2E). Beyond that, the EEF notably influences the alignment of NFA molecules in both neat and blend films. For the control BTP-eC9 film, we observed distinct out-of-plane (OOP) π-π stacking diffraction (010) at 1.87 Å^−1^ (*d* ≈ 3.4 Å) and in-plane (IP) (100) diffraction at 0.41 Å^−1^ (*d* ≈ 15.3 Å) (Fig. 2F and Table 2). In contrast, EEF-treated BTP-eC9 films show a lamellar stacking diffraction (200) at 0.34 Å^−1^ (*d* ≈ 18.5 Å) in the IP direction. These lamellar peaks correspond to the packing between the nearest and next-nearest end-group stackings of Y-series NFA molecules (*3*, *28*). This stacking, primarily along the conjugated backbone, forms a 3D fiber network. The crystal coherence length (CCL) for the next-nearest end-group stacking is 188.50 Å, emphasizing the extended structure of the BTP-eC9 fibrous network. This enhancement and the emergence of a fiber-like network are attributed to EEF-induced long-range ordered end-group stacking, consistent with J-aggregation seen in red-shifted absorption spectra. Remarkably, this ordered 3D network stacking is retained in the EEF-conditioned PM6:BTP-eC9 blend. In EEF-treated PM6:BTP-eC9 films, a unique backbone diffraction pattern appears at 0.60 Å^−1^ (*d* ≈ 10.5 Å) in OOP directions. This diffraction, subtle in pristine films, is linked to the extended π-π stacking distance across the NFA molecules sliding plane (*28*). The extended sliding π-π stacking in NFA fibrous crystals, with a CCL of 62.83 Å, boosts the delocalized transport of π electrons along the conjugated structure in the OOP direction.

**Table 2.** Detailed morphological parameters in controlled and EEF-coated PM6:BTP-eC9.

Samples		Peak (Å^−1^)	*d* spacing (Å)	FWHM (Å^−1^)	CCL (Å)
BTP-eC9 π-π peak, (010)					
BTP-eC9	Control	1.87	3.36	0.37	15.28
	EEF coating	1.86	3.38	0.36	15.71
PM6:BTP-eC9	Control	1.85	3.40	0.34	16.63
	EEF coating	1.84	3.41	0.32	17.67
BTP-eC9 π-π peak, (010), longer distance π-π stacking					
PM6:BTP-eC9	Control	–	–	–	–
	EEF coating	0.60	10.47	0.09	62.83
BTP-eC9 Lamellar peak, (100), next-nearest end-group stacking					
BTP-eC9	Control	–	–	–	–
	EEF coating	0.34	18.48	0.03	188.50
PM6:BTP-eC9	Control	–	–	–	–
	EEF coating	0.34	18.48	0.04	141.37

The role of EEF on the optimizing molecular crystallinity in photovoltaic blends is also demonstrated by the GISAXS pattern (fig. S27 and text S7). Notably, the acceptor domain sizes of the blends remain similar, with dimensions 20 nm for control blend and 21 nm for EEF-coated blend (*6*). These sizes fall within the range conducive to efficient exciton dissociation. However, the intermixing domain size of the EEF-regulated PM6:BTP-eC9 blend is significantly downsized (28 nm for control blend and 9 nm for the EEF-coated blend), which is expected to suppress charge recombination and facilitate the charge collection from this region (the data are summarized in table S10).

### In situ investigations of the EFF coating

**Fig. 3.**
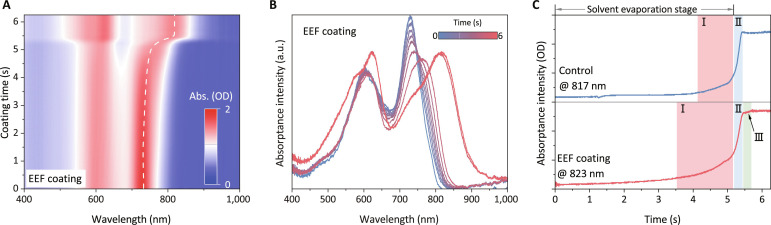
In situ investigations of EEF-enabling formation of highly crystalline during film deposition. (**A**) In situ 2D UV-visible absorption profiles of EEF-coated PM6:BTP-eC9 (*o*-xy) blend during blade coating. (**B**) The corresponding 1D spectra of EEF coating. (**C**) Time evolutions of absorptance intensity for BTP-eC9 in blends during the film fabrication processes, with the tracing wavelength of the acceptor absorption peak of the final solid film. For the blade-coated blend, the crystallization process includes nucleation (2.0 s) and crystal growth (0.5 s). For EEF coating, the crystallization process includes EEF-modulated nucleation (4.0 s), crystal growth (0.5 s), and fine-tuning (0.6 s) stages.

With the understanding of EEF enhanced molecular alignment in solid films, we now delve into the intricate details of how the electric field influences nucleation and crystal growth during the wet coating process of active layers. In situ UV-Vis absorption spectroscopy (Fig. 3A) segments the film-coating process into three stages (Fig. 3B, figs. S34 and S35, and text S8): (I) crystalline nucleation with solvent evaporation, (II) rapid crystal growth, and (III) crystal orientation modification (Fig. 3C, specific to EEF coating) (*5*, *29*). Notable differences in the first and third stages are evident in EEF-coated BHJs compared to control blends. The pre-crystallization process, triggered by the electric field interaction with molecular dipoles, begins earlier, setting the stage for acceptor molecule arrangement (*5*). Additionally, the electric field induces a new stage of crystal orientation refinement in the solid film. This extended molecular organization enhances crystallinity and fibrous morphology, as GIWAXS and AFM patterns confirm. The third stage, akin to thermal annealing, yields comparable results, explaining the satisfactory photovoltaic parameters in annealing-free devices. EEF contribution to active layer crystallinity during blade coating is evident. The EEF modulatory force during solvent evaporation facilitates molecular assembly, fostering an ordered structure that boosts device performance. Crucially, substituting EEF for thermal annealing cuts print preparation time and energy consumed, advancing OSC industrial production (fig. S36).

### Exciton and charge dynamics

**Fig. 4.**
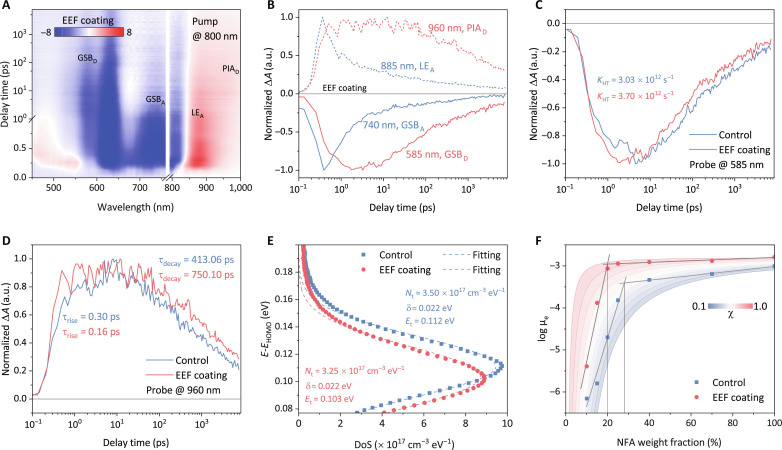
Charge dynamics and long-term operational stability. (**A**) 2D TA data recorded from EEF-coated PM6:BTP-eC9 blend excited by an 800-nm pulsed beam. (**B**) Decay dynamics probed at 585, 740, and 885 nm from the EEF-processed PM6:BTP-eC9 film. (**C**) The HT processes in controlled and EEF-processed PM6:BTP-eC9 films. (**D**) Polaron lifetimes in controlled and EEF-processed PM6:BTP-eC9 films. (**E**) DoSs of the HOMO energy levels of blended films and corresponding Gaussian fitting results. (**F**) Transport data as a function of NFA weight fraction and fitting curves adopted by an electron percolation model with the corresponding percolation threshold.

Femtosecond resolved transient absorption spectroscopy (TAS) was used to provide further insights into how EEF optimization of the active layer microstructure impacts exciton dynamics and charge generation processes. Here, 800-nm excitation pulses were used to individually excite the acceptors in the blends to obtain a hole transfer (HT) signal in the visible wavelengths and a photo-induced absorption band in the near-infrared (NIR) wavelengths. Figure 4A and fig. S37 depict the TAS patterns of PM6:BTP-eC9-based films at visible and NIR wavelengths. With the decay of the ground state blenching (GSB) and singlet exciton signals of acceptor at 740 and 885 nm, the bleach signals of PM6 at 585 nm emerge in the TAS (Fig. 4B). The GSB decay process of the photoexcited BTP-eC9 aligns well with the rise of PM6 GSB, confirming the HT process from the NFA to the donor (*10*). The EEF-coated blend demonstrates an accelerated decay of acceptor localized exciton (LE) at 885 nm, indicating enhanced exciton dissociation. Biexponential decay analysis of LEs revealed that EEF-coated films exhibit a notably faster rate and higher ratio of exciton dissociation compared to control films (increasing from 74% to 83%, table S11) (*10*, *28*). The efficiency of exciton dissociation is significantly influenced by the CCL of the NFA fiber induced by the EEF within the blend. Exponential fitting of PM6 GSB yields a hole transfer rate (*K*_HT_) of 3.03 ps^−1^ for the control film and 3.70 ps^−1^ for the EEF-coated film (Fig. 4C and table S12). In addition, the prolongation lifetime of the photo-induced absorption of donor charge at 960 nm suggests that the EEF-modulated micromorphology contributes to the suppression of bimolecular recombination of the free polarons (Fig. 4D and table S13). Time-resolved photoluminescence spectroscopy corroborates higher acceptor exciton dissociation rate and lower exciton recombination, consistent with TAS results (figs. S38 to S40, table S14, and text S9).

We also investigated how the EEF influences the charge dynamics and recombination in OSCs by inducing NFA fibers with favorable percolation threshold and crystallinity. Density of states (DoS) analysis showed a reduced density of carrier energy defects (*N*_t_) near the HOMO and a DoS center (*E*_t_) closer to the HOMO, indicating less energy loss due to defect levels (Fig. 4E, fig. S41, and text S10). This reduction suggests that the EEF mitigates nonradiative recombination in the regulated film by enhancing crystallinity, as evidenced by the increased *V*_OC_ in EEF-coated devices. To examine how EEF-induced NFA fibers affect charge dynamics in OSCs, we analyzed the percolation threshold (χ) of NFA in the BHJ blend films (Fig. 4F, fig. S42, and table S15). The conjugated structure of EEF-induced NFA fibers yielded a χ of 0.6 to 0.7, implying stable and favorable electron mobility in the high donor:acceptor ratio blended phase, which helps minimize recombination and increase the *J*_SC_s of OSCs (text S11). To understand the suppression of carrier recombination by the favorable percolation threshold induced by NFA fibers, transient photovoltage/current (TPV/TPC) testing was used to investigate transport and recombination parameters of photogenerated carriers (fig. S43). The favorable percolation threshold of NFAs facilitates the high-speed transport of photogenerated carriers within the active layer, thereby suppressing charge recombination and efficient extraction, as we obtain from TPV and TPC lifetime.

### Vertical distribution of donor and acceptor fractions

**Fig. 5.**
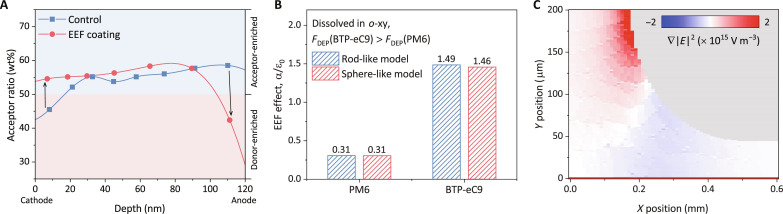
Vertical composition profiles of thin films. (**A**) Film depth–dependent composition profiles extracted from FLAS of the *o*-xy processed films. The depth of 120 nm is the near-substrate side of the active layer, and 0 nm is the far-substrate side. (**B**) Estimation of the term α/ɛ_0_ for working frequency (200 Hz) and two solvents with different polarities, indicating their influence in DEP. (**C**) Distribution of the square of the electric field gradient in the coating process.

In addition to the molecular packing behavior, the vertical distribution gradient of donor and acceptor materials within active layers has been demonstrated to be crucial factors in determining the photovoltaic performances of OSCs (*30*). The film depth–dependent light absorption spectroscopy (FLAS) has been demonstrated to be an effective method to characterize distribution of acceptor:donor ratios across the depths of the photoactive layer. The FLAS reveals film depth–dependent composition distributions for the control and EEF-coated films (Fig. 5A and figs. S44 and S45). From this result, we can see that EEF leads to the formation of favorable vertical gradient with more acceptor relatively enriched near the cathode interface and reduced acceptor ratio near the anode interface. This increased fraction of acceptors in the near-cathode leads to the red shift of absorption peaks as we obtained above (*15*). Meanwhile, we also investigated the vertical component distribution pattern of the films when highly polar CF was used as the processing solvent, and the results and discussion are shown in figs. S46 and S47 and text S12. Additionally, we characterized the built-in potentials of the control (0.81 V) and EEF-coated (0.85 V) devices (fig. S48) and the elevated values are attributed to the optimization of the donor:acceptor distribution throughout the devices, which is consistent with previous observations (*15*, *31*). Besides this, the suppressed device dark current is also consistent with the improved vertical component gradient of acceptor (fig. S48). All these evidences support the idea that EEF-coated devices have better vertical component gradient, which is obviously beneficial for the device performance.

To reveal the origin of improved vertical distribution of acceptor upon EEF coating, we considered dielectrophoresis (DEP) of organic molecules as a probable cause. DEP, as a well-developed theory, reveals the movement of suspended particles relative to the solvent, induced by polarization forces generated by an inhomogeneous electric field (*32*, *33*). The DEP force acting on such particle can be described as ${\vec{F}}_{\mathrm{DEP}}=\frac{1}{2}\alpha \nu \cdot \nabla {|E|}^{2}$ , where α is its polarizability of the particle, ν denotes the particle volume, and ∇∣*E*∣^2^ represents the squared gradient of the applied electric field (*32*). Figure 5B illustrates how the EEF effect (α/ɛ_0_ with ɛ_0_ = 8.85 × 10^−12^ F m^−1^ being the electrical permittivity of vacuum) varies when different solvents are used (see figs. S49 to S51 and text S13 for relevant details). PM6 and BTP-eC9 molecules show positive polarization values in the *o*-xy solvent, and therefore, the nonuniform electric field generates DEP forces pulling PM6 and BTP-eC9 molecules toward the high electric field region at nanoscale velocities. Notably, the force acting on BTP-eC9 molecules is significantly stronger than that on PM6 leading to the drift of the acceptor molecule toward the upper part of the meniscus as depicted in Fig. 5C and deposited on the surface of the solid film. The increase of acceptor molecule concentration at the surface of the meniscus induces an earlier nucleation process, as demonstrated in the in situ film formation dynamics. Meanwhile, this result is in agreement with our observation in the vertical component gradient that the acceptor molecules are more distributed near the cathode of the OSCs. However, the DEP force provided by EEF in films using CF as a processing solvent is reversed from the process described above, leading to another component distribution pattern that results from the difference in polarity between CF and organic molecules (see text S13 for details).

## DISCUSSION

We demonstrate the potential of the EEF-coating approach to fine-tune the morphology of photoactive layers in the wet coating process. With the EEF treatment, a record high efficiency of 18.6% was achieved in the open-air printing process with eco-friendly solvent. Such performance enhancement in EEF-coated devices can be attributed to the formation of a fibrous structure of acceptor domains and desirable vertical phase distribution. Within our study, this unique nonchemical EEF-coating approach can be also effective for a wide range of BHJ systems. This work demonstrates the potential of the EEF-coating method in the field of OSCs through device preparation and mechanism analysis, and lays the foundation for broader industrial production in the field of organic electronic devices.

## MATERIALS AND METHODS

### Materials

PM6 (poly((4,8-bis(5(2-ethylhexyl)-4-fluoro-2-thienyl)benzo[1,2-b:4,5-b′] dithiophene-2,6-diyl)-2,5-thiophenediyl(5,7-bis(2-ethylhexyl)-4,8-dioxo-4H,8H-benzo[1,2-c:4,5-c′]dithiophene-1,3-diyl)2,5-thiophenediyl)), D18 (poly(dithieno[3,2-e:2′,3′-g]-2,1,3-benzothiadiazole-5,8-diyl(4-(2-butyloctyl)-2,5-thiophenediyl)(4,8-bis(5-(2-ethylhexyl)-4-fluoro-2-thienyl)benzo[1,2-b:4,5-b′]dithiophene-2, 6-diyl)(3-(2-butyloctyl)-2,5-thiophenediyl))), ITIC (2,2′-((6,6,12,12-tetrakis(4-hexylphenyl)6,12-dihydrodithieno[2,3-d:2′,3′-d′]-s-indaceno[1,2-b:5,6-b′]dithiophene-2,8-diyl)bis(methylidyne(3-oxo-1H-indene-2,1(3H)diylidene)))bis(propanedinitrile)), Y6 (2,2′-((2Z,2′Z)-((12,13-bis(2-ethylhexyl)-3,9diundecyl-12,13-dihydro-[1,2,5]thiadiazolo[3,4-e]thieno[2′′,3′′:4′,5′]thieno[2′,3′:4,5]pyrrolo[3,2-g]thieno[2′,3′:4,5]thieno[3,2-b]indole-2,10-diyl)bis(methaneylylidene))bis(5,6-difluoro-3-oxo-2,3-dihydro-1H-indene-2,1-diylidene))dimalononitrile), BTP-eC9 (2,2′-((2Z,2′Z)-((12,13-bis(2-butyloctyl)-3,9-dinonyl12,13-dihydro-[1,2,5]thiadiazolo[3,4-e]thieno[2′′,3′′:4′,5′]thieno[2′,3′:4,5]pyrrolo[3,2-g]thieno[2′,3′:4,5]thieno[3,2-b]indole-2,10-diyl)bis(methaneylylidene))bis(5,6-dichloro-3-oxo-2,3-dihydro-1H-indene-2,1-diylidene))dimalononitrile), and BTP-eC9-2Cl (2,2′-((2Z,2′Z)-((12,13-bis(2-butyloctyl)-3,9-dinonyl-12,13-dihydro-[1,2,5]thiadiazolo[3,4-e]thieno[2″,3″:4″,5″]thieno[2″,3″:4,5]pyrrolo[3,2-g]thieno[2″,3″:4,5]thieno[3,2-b]indole-2,10-diyl)bis(methaneylylidene))bis(6-chloro-3-oxo-2,3-dihydro-1H-indene-2,1-diylidene))dimalononitrile) were purchased from Solarmer Materials Inc. CF and *o*-xylene were purchased from Sigma-Aldrich LLC. All other reagents and chemicals were purchased from commercial sources and used without further purification.

### EEF-coating setup

The DC voltage source with adjustable voltage is used as the control terminal to adjust the output pulse voltage (5 to 2 kV) of the operational amplifier. AFG1000 Arbitrary/Function Generator (TEKTRONIX, INC.) was used as a device for generating voltage pulses (200 Hz), which was connected to the signal input terminal of the operational amplifier circuit. Automatic film applicators ZAA2300.H, Proceq Trading (Shanghai) Co. Ltd., for use with heatable plate provided an accurate and reproducible application of coating films. A 150 mm × 150 mm × 0.1 mm copper plate served as the positive end of the voltage pulse, sandwiched between two ceramic plates placed on the heatable plate (to ensure that it was not shorted) as the terminal of the voltage pulse. All active layers used were prepared by placing a suitable substrate on the voltage pulse terminal. The zero potential of the voltage pulse is connected to manual height adjustable aluminum blade ZUA 2000, Proceq Trading (Shanghai) Co. Ltd. The principles of experimental setup design, electric field distribution, selection of electric field strength, and a preliminary discussion of the effects of electric fields on organic molecules are provided in figs. S1 to S6, table S1, and texts S1 to S3.

### Photovoltaic device fabrication and measurement

Organic photovoltaic devices with structure of indium tin oxide (ITO)/Poly(3,4-ethylenedioxythiophene) polystyrene sulfonate (PEDOT:PSS)/Active-layer/PNDIT-F3N/Ag were fabricated. Patterned ITO substrates (R_sheet_ = 15 ohms per square) were cleaned in sequence in detergent, deionized water, acetone, and isopropanol in an ultrasonic bath for 20 min, respectively. PEDOT:PSS solution (PEDOT:PSS:deionized water is 1:1, v/v) was spin-coated onto the ultraviolet-ozone–treated ITO substrates, followed by annealing at 150°C for 10 min under ambient conditions. Photoactive blends of PM6:BTP-eC9 (1:1.1, m/m) and PM6:D18:BTP-eC9 (0.8:0.2:1.1, m/m/m) with a polymer concentration of 10 mg ml^−1^ were dissolved in *o*-xylene and stirred at 80°C for 4 hours. 1,8-Diiodooctane with a volume ratio of 0.3% was added as an additive to the main solvent. Blended solutions were blade-coated onto the top of the PEDOT:PSS layer at 25 mm s^−1^ and a blade-substrate gap of 150 μm at 90°C for *o*-xy processed (for thermal annealing required devices, followed by further annealing at 100°C for 10 min in the N_2_ glovebox). For CF-processed devices, PM6:BTP-eC9 (1:1.1, m/m) with a polymer concentration of 10 mg ml^−1^ was dissolved and stirred at 40°C for 2 hours, and 1,8-Diiodooctane with a volume ratio of 0.3% was added as an additive to the main solvent. Then, the PNDIT-F3N (0.5 mg ml^−1^ in methanol with 0.5% acetic acid, v/v) was spin-coated onto the active layer at 3000 rpm for 30 s, followed by thermal deposition of Ag (100 nm) top electrode under high vacuum (<2.5 × 10^−4^ Pa) to fabricate devices with an effective area of 0.063 cm^2^ and 1 cm^2^. For CF-processed inverted devices, the structure is ITO/ZnO/Active-layer/MoO_3_/Al, where ZnO was spin-coated on the substrate by a precursor solution in air at 3500 rpm and heated at 200°C for 2 hours. Both MoO_3_ and Al were obtained by physical vapor deposition. The cells were illuminated through aperture areas of 0.063 cm^2^ and 1 cm^2^. The measurement was performed in a N_2_-filled glovebox at 25°C. The AM 1.5 G illumination was simulated by a simulator and calibrated using a NIM (National Institute of Metrology, China)–certified monocrystalline Si solar cell. There was almost no variation of the performance with or without aperture. The *J*-*V* curves were measured under AM 1.5 G illumination of 100 mW cm^−2^ by a Keithley 2400 source meter unit. The 7-SCSpec system (Sofn Instrument Co. Ltd.) was used to investigate the EQE spectrum of organic photovoltaic devices.

### Optoelectronic characterization

UV-Vis absorption spectra of the pristine and blend films were measured by a UV-Vis-NIR spectrophotometer (Cary 5000, Agilent Technologies Inc.) under ambient conditions. Samples were fabricated on quartz substrates.

### AFM image

AFM measurements were performed on a Bioscope Resolve AFM (Bruker) in a tapping mode under ambient conditions.

### GIWAXS/SAXS measurement

GIWAXS/SAXS measurements were performed at the Shanghai Synchrotron Radiation Facility BL16B1 beamline under ambient conditions. X-rays have a wavelength of 1.23984 Å and sample detector distance was calibrated by a silver behenate (AgBH). The angles of grazing incidence for GIWAXS/SAXS measurements are 0.10°/0.15°. Samples were prepared on silicon/poly(3,4-ethylenedioxythiophene) polystyrene sulfonate substrates using identical blend solutions and methods as those used in photovoltaic device fabrication.

### Film depth–dependent light absorption spectroscopy

The FLAS was acquired upon a film depth–dependent light absorption spectrometer (PU100, Puguangweishi Co. Ltd.). In situ soft plasma etching at low pressure (10 to 20 Pa) was used to extract the depth-resolved absorption spectrum for the organic active layer. Beer-Lambert’s law was utilized to fit the FLAS results, which were subsequently utilized to fit the exciton generation contour upon a modified optical matrix-transfer approach.

### In situ characterization

Xenon lamps were used as light sources to obtain in situ transmission absorption spectrum from a Maya2000 Pro (Ocean Insight) spectrometer for the blade-coating process.

### Photoluminescence spectroscopy

The steady-state PL spectra of the pristine and blend films were acquired through a confocal optical microscope (Nanofinder FLEX2, Tokyo Instruments Inc.). All of the PL spectra were measured using a charge-coupled device sensor (DU420A-OE, Andor, Oxford Instruments). The excitation wavelength was fixed at 400 nm with a power of 5 μJ cm^−2^. The sample temperature was controlled by a Model 325 Cryogenic Temperature Controller (Lake Shore Cryotronics Inc.). Samples were fabricated on quartz substrates and were encapsulated in a nitrogen glovebox with low-temperature epoxy resin.
