# Supplementary material for: Using an external electric field to tune active layer morphology enabling high-efficiency organic solar cells via ambient blade coating
Source: Sci Adv. 2024 Jun 28;10(26):eado5460. doi: 10.1126/sciadv.ado5460 (PMC11212706; doi:10.1126/sciadv.ado5460)
Supplement: Supplementary file 1 — Text S1 to S13 Figs. S1 to S51 Tables S1 to S15 [file sciadv.ado5460_sm.pdf]

Science Advances
AAAS

Supplementary Materials for

Using external electric field to tune active layer morphology enabling high-efficiency organic solar cells via ambient blade coating

Fengzhe Cui *et al.*

Corresponding author: Xiaotao Hao, haoxt@sdu.edu.cn; He Yan, hyan@ust.hk; Hang Yin, hyin@sdu.edu.cn

Sci. Adv. **10**, eado5460 (2024)
DOI: 10.1126/sciadv.ado5460

This PDF file includes:

Text S1 to S13
Figs. S1 to S51
Tables S1 to S15

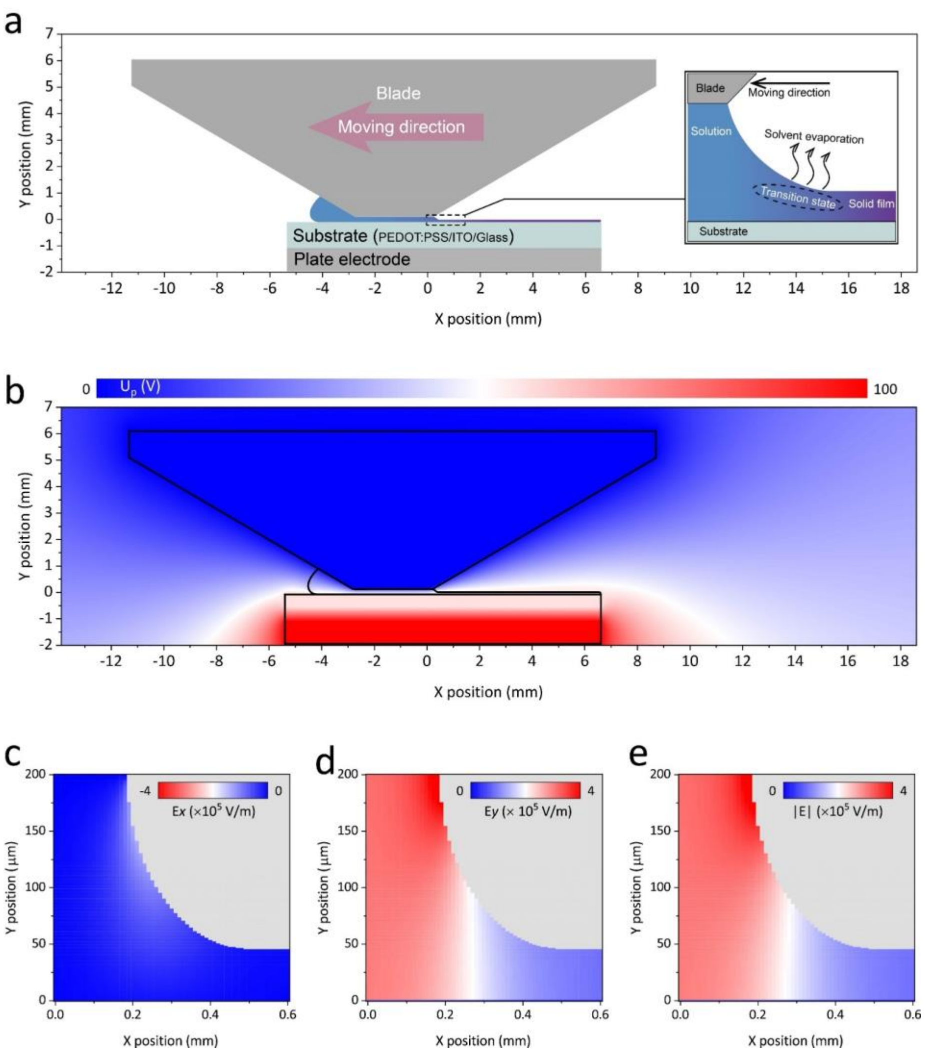

Figure S1 │ Calculated electric field distribution pattern. a, Details of the model used in the electric field strength calculation. **b**, Calculated electric field distribution pattern. **c**, Horizontal fraction of electric field strength in the meniscus. **d**, Vertical fraction of electric field strength in the meniscus. **e**, Total value of electric field strength in the meniscus

Supplementary text S1:

The electric field distribution is modeled and calculated using COMSOL Multiphysics 5.5. The model used for the calculation of the electric field distribution at peak voltage has a relative dielectric value of 1 for air, a value of +∞ for indium tin oxide, a value of +∞ for plate electrode, a value of +∞ for aluminum blade, a value of 7.0 for anodized aluminum oxide protective coating for blade, a value of 4.2 for glass substrate, a value of 2.5 for solution.

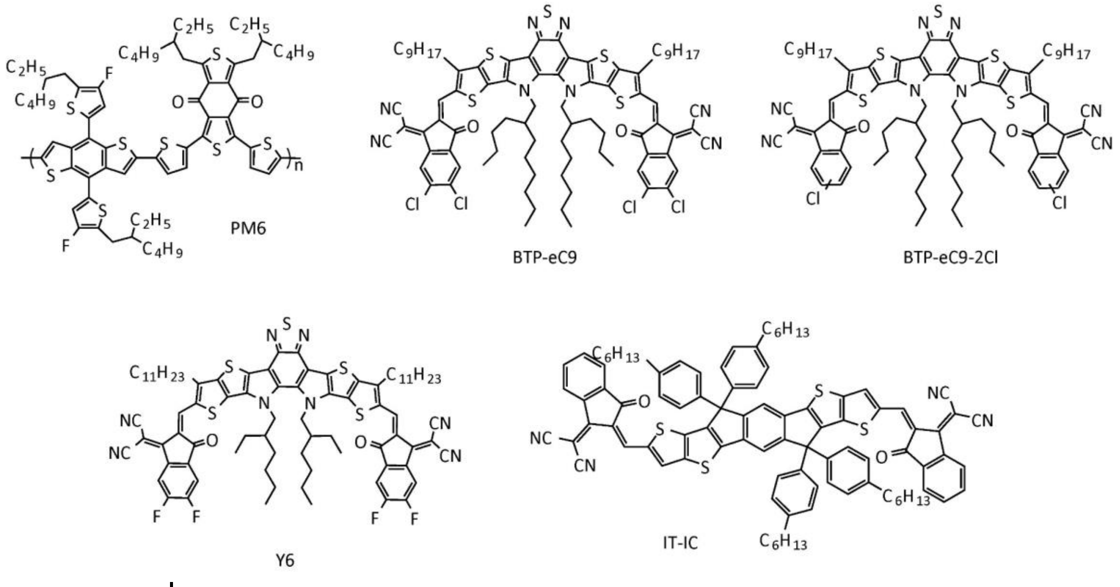

Figure S2 │ Chemical structures. Chemical structures of donor PM6 and NFA materials utilized.

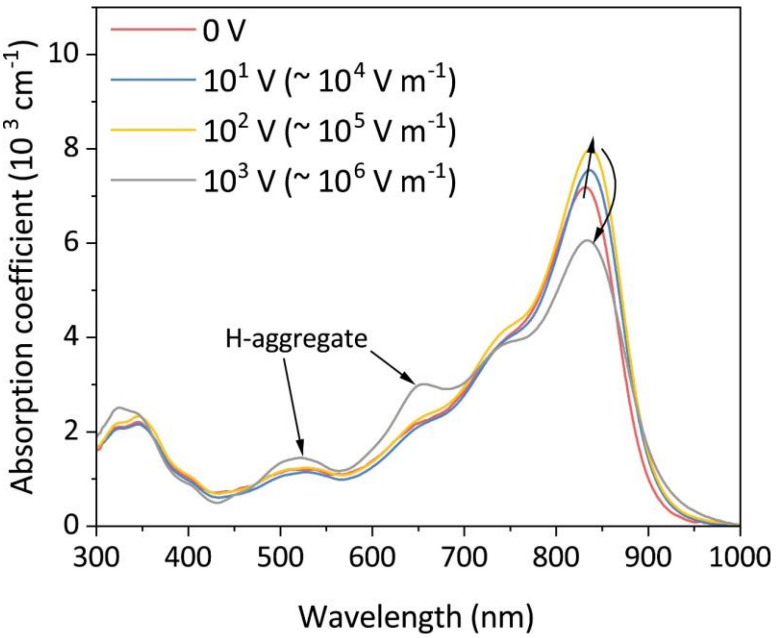

Figure S3 │ Absorption coefficient of *o*-xylene processed BTP-eC9. Variation of the absorption coefficients of BTP-eC9 films prepared at different orders of electric field intensities. With the enhancement of electric field intensity, the J-type aggregation absorption was gradually enhanced, but when the electric field intensity reached ~10^6 V m^{-1}, too much J-type aggregation was transformed into H-type aggregation absorption thus unfavorable to the harvesting of photons. Therefore, the electric field strength of ~10^5 V $m^{-1}$was selected for conducting the subsequent study.

Supplementary text S2:

We screened according to the absorption coefficients of BTP-eC9 coated at different electric field intensities. For the absorption spectrum of the control BTP-eC9, the absorption at 820 nm is the absorption band of J-type aggregation, the absorption band of monomer at 750 nm, and the absorption band of H-type aggregation at 650 nm and 550 nm(*4, 5*). When the electric field strength of 10^4-10^5 V m^{-1} was used for electric field coating, the absorption bands of J-type aggregates gradually enhanced, indicating the formation of more J-type aggregates, which was also verified in the subsequent GIWAXS and GISAXS. However, when the electric field intensity using electric field coating is increased to 10^6 V m^{-1}, the excessive torque leads to the formation of more

core-to-core H-aggregates, which is not only unfavorable for photon harvesting, but also for exciton diffusion and charge transport. As a result, we finally chose 10^5 V m^{-1} as a suitable electric field strength to continue the related study.

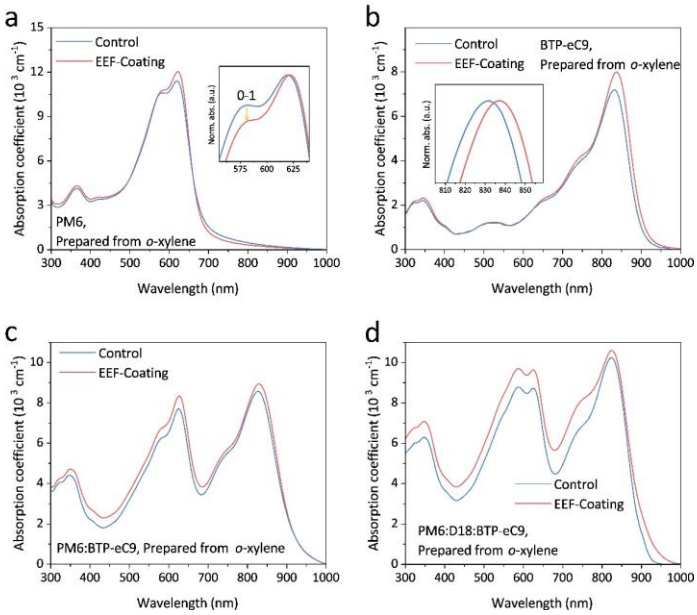

Figure S4 │ Absorption characteristics. a, *o*-xylene processed PM6, **b**, *o*-xylene processed BTP-eC9, **c**, *o*-xylene processed PM6:BTP-eC9, and **d**, *o*-xylene processed PM6:D18:BTP-eC9 UV-vis absorption curves.

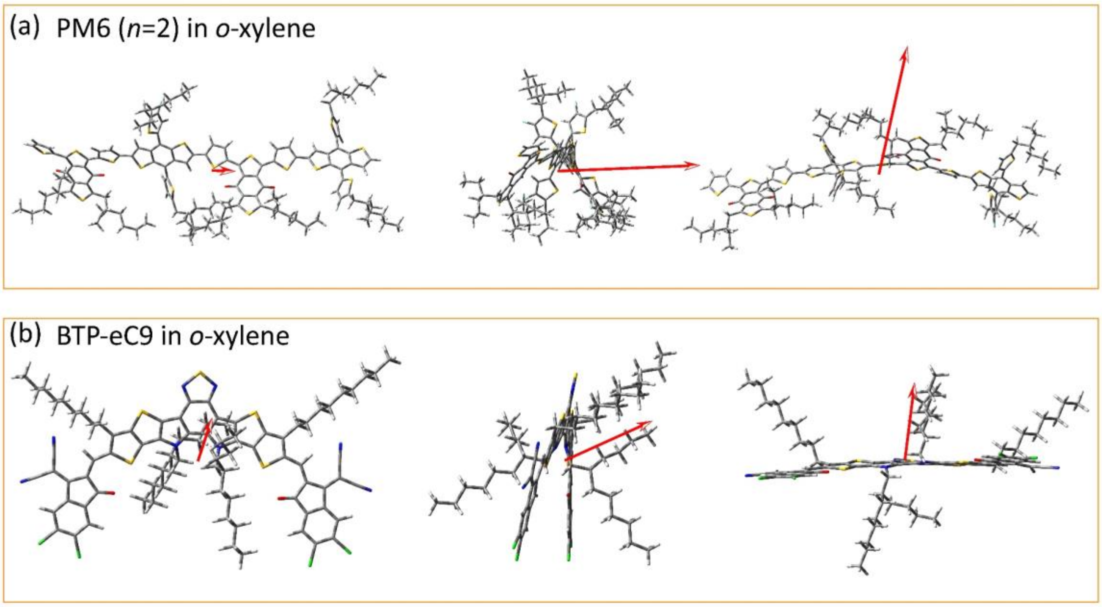

Figure S5 │ Calculated molecular dipole moment. a, Calculated molecular dipole moment obtained by Gaussian 09 performed at the B3LYP/6-31G(d,p) level of PM6. **b**, Calculated molecular dipole moment of BTP-eC9.

Table S1 │ Molecular dipole moments of PM6 and BTP-eC9 when solvation effects are considered.

Molecules	μx (Debye)	μy (Debye)	μz (Debye)	μ (Debye)
PM6 (*n*=2)	1.46	-0.13	4.69	4.91
BTP-eC9	0.27	0.91	2.06	2.27

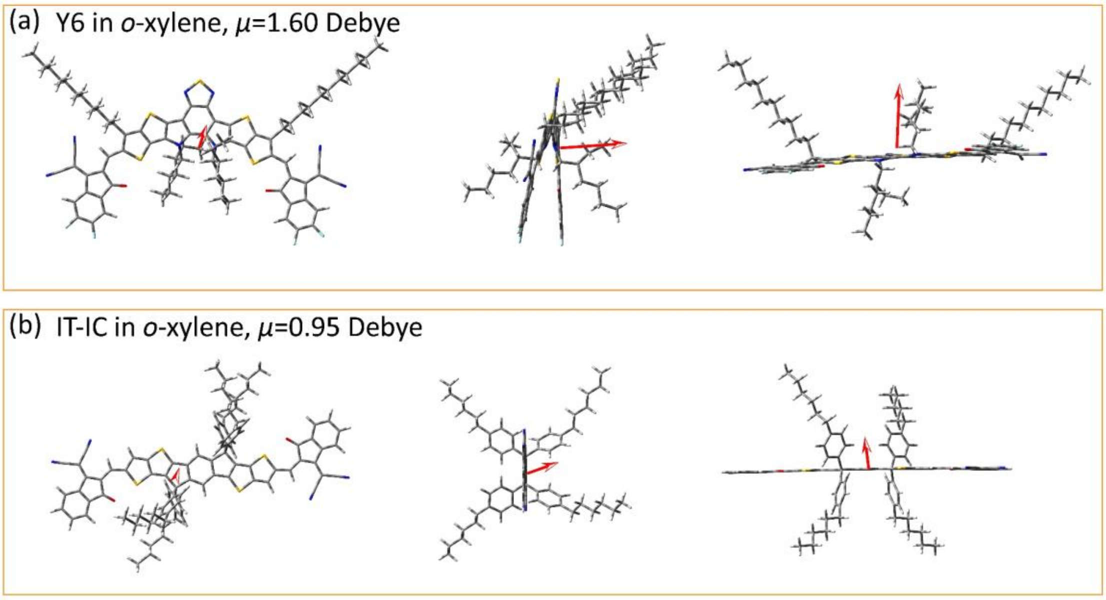

Figure S6 │ Calculated molecular dipole moment. a, Calculated molecular dipole moment obtained by Gaussian 09 performed at the B3LYP/6-31G(d,p) level of Y6. **b**, Calculated molecular dipole moment of IT-IC.

Supplementary text S3:

Electric dipole ($\vec{\mu}$) interactions between donor and acceptor molecules have unique effects on molecular stacking within the active layer. In line with classical electrostatics, when dealing with non-spherically shaped or anisotropic particles, an EEF applies a torque ($\vec{T}$) to the dielectric particle. As shown in the inset of Fig. 1A, this torque tends to align the dipole with the direction of the external E-field and is typically described as $\vec{T} = \vec{\mu} \times \vec{E}$. According to our estimation, a dipole with a moment of 3.0 Debye receives a torque of $1.0\times10^{-15} \sim 1.0\times10^{-13}$ N Å at an angle of 45 degrees under an electric

field of $10^3 \sim 10^6$ V m^{-1}. At the B3LYP/6-311G (d, p) level, density generalized function theory calculations taking into account solvation effects are performed by Gaussian 09. The calculated dipole moments are listed in **Table S2**. We find that the orientation of the molecular dipoles of PM6 and BTP-eC9 in solution is approximately parallel to the π-π stacking direction. This finding supports the feasibility of controlling face-on π-π packing using the vertical E-field as described above. As shown in **Fig. S4**, the ultraviolet-visible absorption spectra of PM6 and BTP-eC9 films exhibit red-shifted 0-0 transition peaks and increased 0-0/0-1 intensity ratio under the modulation of EEF. These observed alterations, including the red-shifted transition peaks and the modified ratio of J-type aggregation absorption, are indicative of partially more ordered microstructures within the films. The EEF influence on the molecular dipole effectively induces optimized molecular orientation in the transition state and promotes partially ordered microstructures in the film, thereby enhancing the films absorption coefficient.

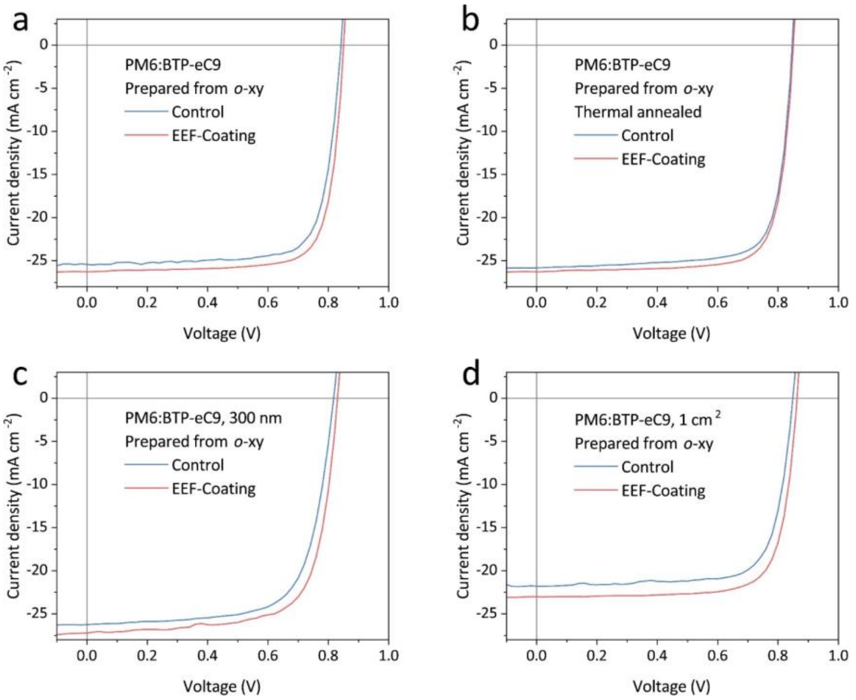

Figure S7 │ Photovoltaic performance. *J-V* curves of the OSCs based on **a**, PM6:BTP-eC9 (*o*-xylene) without annealing treatment, **b**, with annealing treatment, **c**, with active layer of 300 nm, and **d**, device area of 1 cm^2 under the illumination of AM 1.5 G 100 mW cm^{-2}.

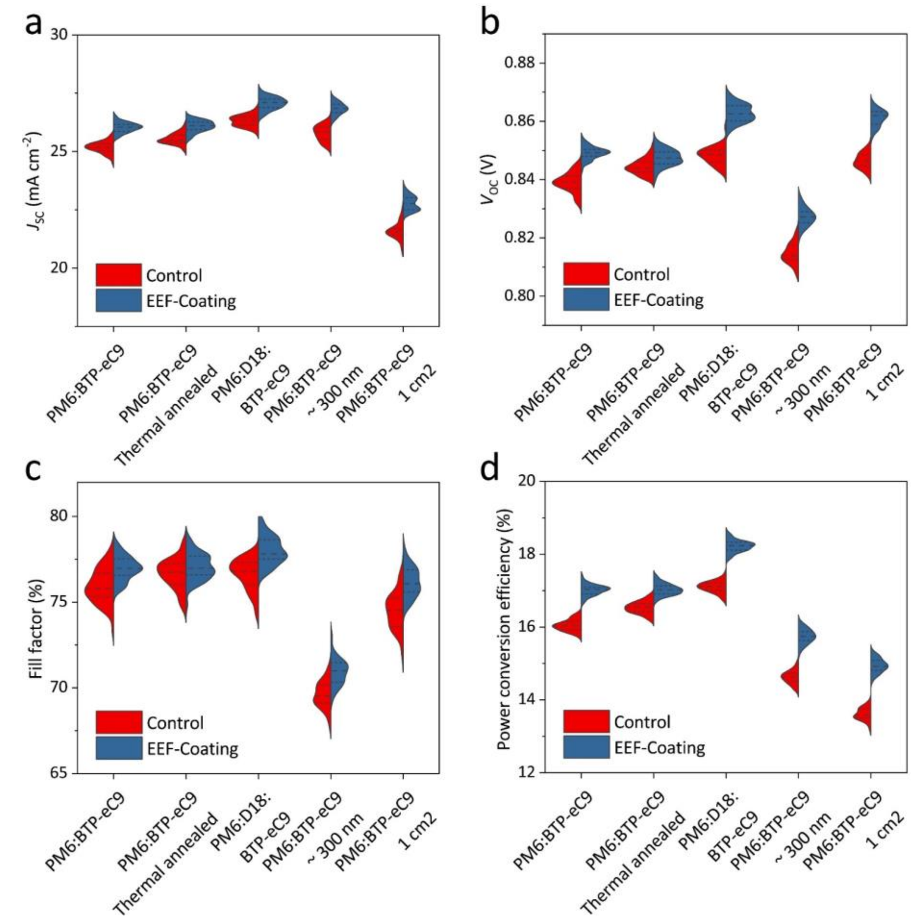

Figure S8 │ Device parameters. Violin plots of **a**, fill factor, **b**, short-circuit current density, **c**, open circuit voltage and **d**, PCE distribution of the different devices (n=50 or n=30).

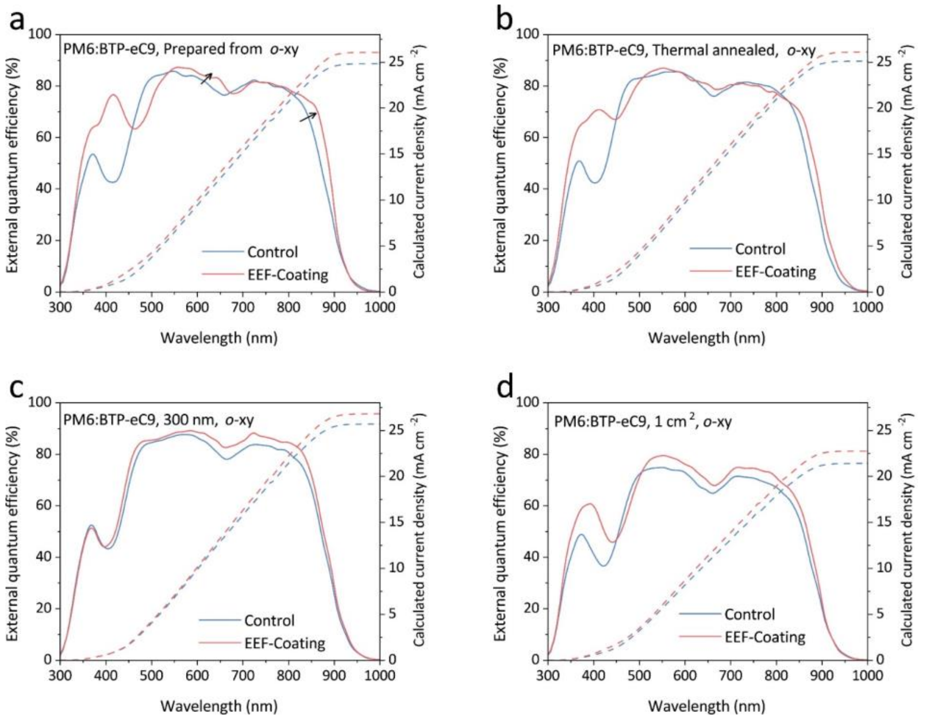

Figure S9 │ External quantum efficiency and integrated current density. EQE curves of the OSCs based on PM6:BTP-eC9 (*o*-xylene) **a**, without additional annealing treatment, **b**, with annealing treatment, **c**, with active layer of 300 nm, and **d**, with device area of 1 cm^2.

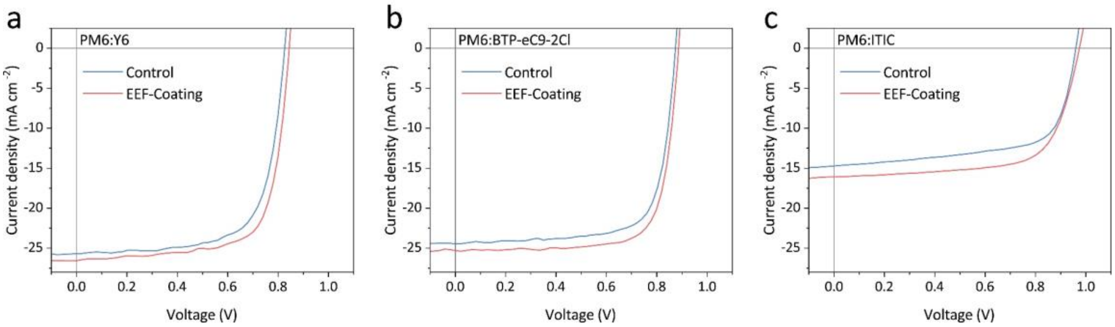

Figure S10 │ Photovoltaic properties. Current density-voltage characteristics of the **a**, PM6:Y6, **b**, PM6:BTP-eC9-2Cl, and **c**, PM6:ITIC devices under simulated air mass 1.5 global illumination at 100 mW cm^{-2}.

Table S2│ The summary of PCEs of the open-air coated OSCs under the illumination of AM 1.5 G.

Samples	J_{sc} (mA cm^{-2})	V_{oc} (V)	FF (%)	PCE (%)	Ref
PM6:BTP-eC9	25.8	0.84	76.0	16.4	Nat. Commun. 12, 4815 (2021).
	26.7	0.84	78.1	17.5	Nat. Commun. 12, 4815 (2021).
	25.2	0.84	75.3	15.9	Nat. Commun. 12, 4815 (2021).
	26.3	0.84	76.4	16.8	Nat. Commun. 12, 4815 (2021).
PM6:BTP-eC9	26.0	0.84	75.7	16.6	Adv. Funct. Mater., 2303403.
PM6:BTR-Cl:BTP-eC9	26.4	0.84	78.2	17.3	Adv. Funct. Mater., 2303403.
FTAZ:IT-M	16.8	0.95	66.6	10.7	Adv. Mater. 30, 1705485 (2018).
PM6:Y6-hu	26.7	0.85	76.5	17.4	Adv. Energy Mater. 13, 2203452 (2023).
	25.8	0.84	74.1	16.0	Adv. Energy Mater. 13, 2203452 (2023).
PTQ10:Y6-C12	21.2	0.86	57.9	10.6	ACS Appl. Mater. Interfaces 14, 57055–57063 (2022).
PM6:Y6-C12	23.8	0.80	56.0	10.6	Adv. Mater. Interfaces 10, 2202156 (2023).
PM6:BTR-Cl:Y6	26.7	0.82	71.0	15.6	Adv. Funct. Mater. 33, 2210534 (2023).
PM6:BTR-Cl:CH1007	27.0	0.82	74.1	16.3	Adv. Funct. Mater. 33, 2210534 (2023).
PM6:Y6C12	24.1	0.80	62.0	11.9	Sol. RRL 6, 2200691 (2022).
PM6:L8-BO	25.4	0.87	77.2	17.0	Adv. Mater. 34, 2202659 (2022).
	25	0.87	75.6	16.3	Adv. Mater. 34, 2202659 (2022).
D18:Y6	27.4	0.84	73.9	17.1	J. Mater. Chem. A 10, 13439–13447 (2022).
PM6:Y6	25.7	0.84	68.0	14.7	J. Mater. Chem. A 10, 13439–13447 (2022).
PM6:BTP-eC9	26.3	0.80	73.6	15.5	Mater. Today 55, 46–55 (2022).
PM6:Y6	23.5	0.80	71.3	13.5	J. Phys. Mater. 4, 044016 (2021).
PBDTTT-OFT: PC71BM	15.7	0.71	63.6	7.02	J. Phys. Mater. 4, 044016 (2021).
PBDTTT-OFT: IEICO-4F	22.6	0.67	71.6	10.9	J. Phys. Mater. 4, 044016 (2021).
PM6:Y6	16.3	0.75	72.2	8.8	J. Phys. Mater. 4, 044016 (2021).
PM6:Y6	23.2	0.81	59.2	11.0	ACS Appl. Mater. Interfaces 13, 49096–49103 (2021).
PTQ10:Y6	18.4	0.86	59.8	9.0	ACS Appl. Mater. Interfaces 13, 49096–49103 (2021).
PBDB-T:PC61BM	12.8	0.87	58.0	6.5	ACS Appl. Mater. Interfaces 14, 1568–1577 (2022).
PM6:Y6	24.9	0.8	54.0	10.9	ACS Appl. Mater. Interfaces 14, 3103–3110 (2022).
PDTBT2T-FTBDT:BTP-4F	23.5	0.85	64.5	12.8	Adv. Energy Mater. 12, 2103977 (2022).
	18.5	0.79	41.4	6.0	Adv. Energy Mater. 12, 2103977 (2022).
PTQ10:Y6-BO	25.6	0.87	71.0	15.8	ACS Appl. Mater. Interfaces 14, 13572–13583 (2022).
	25.6	0.86	69.4	15.3	ACS Appl. Mater. Interfaces 14, 13572–13583 (2022).
	25.5	0.85	65.3	14.2	ACS Appl. Mater. Interfaces 14, 13572–13583 (2022).
FBT:PC61BM:PDI	13.4	0.88	68.0	7.9	ACS Appl. Mater. Interfaces 12, 43684–43693 (2020).
PTB7-TH:IEICO-4F	22.0	0.70	67.5	10.4	J. Mater. Chem. A 8, 10318–10330 (2020).
PTB7-Th:EH-IDTBR:T2-OEHRH	18.1	1.04	65.0	12.1	Sol. RRL 4, 2000246 (2020).
PV2000:PCBM	16.3	0.81	74.0	9.8	Sol. Energy Mater. Sol. Cells 202, 110064 (2019).
PM6:IT-4F	20.8	0.88	72.0	13.2	J. Mater. Chem. A 7, 22265–22273 (2019).
D18:Y6	27.0	0.85	75.1	17.2	Advanced Energy Materials 13, 2203496 (2023).
PTzBI:N2200	14.9	0.84	66.7	8.4	Nano Energy 59, 277–284 (2019).

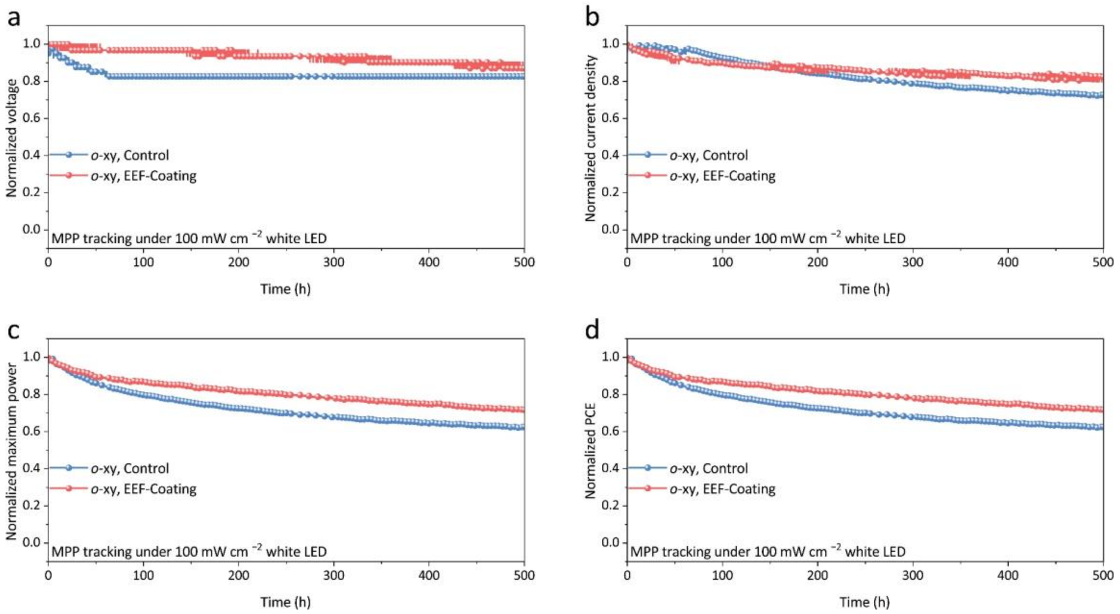

Figure S11 │ Maximum power point tracking under 100 mW cm^{-2} white LED. a, Voltage, **b**, current density, **c**, maximum power, and **d**, PCE stability test of encapsulated OSCs based on PM6:BTP-eC9 (*o*-xy) with conventional structure and initial PCEs of 15.8% and 16.9% respectively, stored in open-air under the illumination of a 100 mW cm^{-2} white LED.

Supplementary text S4:

Device operational stability is a concern that is used to evaluate the commercialization benefits. We compared the operational stability of the control and EEF-coated PM6:BTP-eC9 devices with maximum power point tracking under the continuous 100 mW cm-2 white LED lighting condition for 600 hours. PCEs expressed as a function of the LED light-soaking time are exhibited in **Fig. S10**. The EEF-coated system using o-xylene as the solvent maintained a more stable normalized PCE (retaining 72.0% of its initial PCE), while the control system retained only 62.2% of its initial PCE.

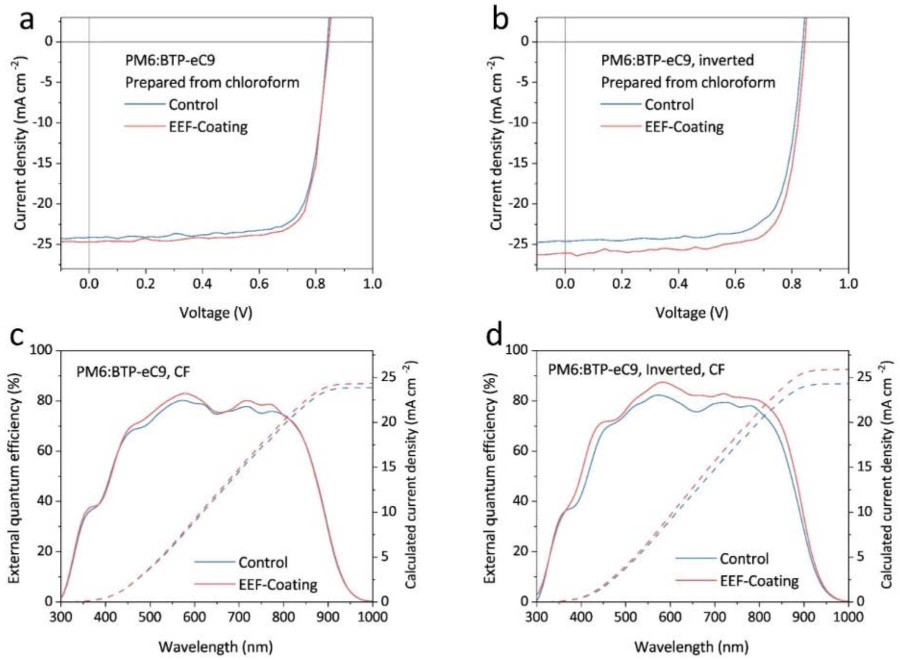

Figure S12 │ Photovoltaic properties. a, **b,** Current density-voltage (*J*-*V*) characteristics of the chloroform processed PM6:BTP-eC9 devices under simulated air mass 1.5 global illumination at 100 mW cm^{-2}. **c**, **d,** Corresponding external quantum efficiency spectra (solid lines) and integrated J_{SC}s (scatters) of the chloroform processed PM6:BTP-eC9 devices.

Table S3 │ Summary of photovoltaic parameters of the PM6:BTP-eC9 devices prepared in different methods.

Samples	EEF	J_{sc} (mA cm^{-2})	$J_{cal.}$ (mA cm^{-2}) [a]	V_{oc} (V)	FF (%)	PCE (%)[b]
PM6:BTP-eC9	w/o	24.1	23.9	0.841	77.7	15.7 (15.4±0.2)
(CF, conventional)	w/	24.7	24.3	0.845	77.9	16.3 (15.9±0.2)
PM6:BTP-eC9	w/o	24.6	24.3	0.839	74.8	15.4 (15.2±0.2)
(CF, inverted)	w/	26.1	25.9	0.846	75.7	16.7 (16.3±0.2)

[a] Integrated current densities from external quantum efficiency curves.

[b] Average values with standard deviation were obtained from 10 devices.

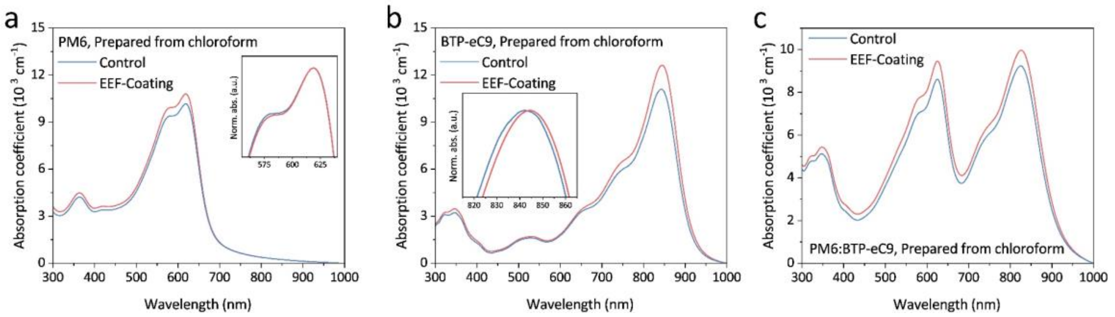

Figure S13 │ Absorption characteristics. The UV-vis absorption curves of **a**, chloroform processed PM6, and **b**, chloroform processed BTP-eC9, and **c**, chloroform processed PM6:BTP-eC9.

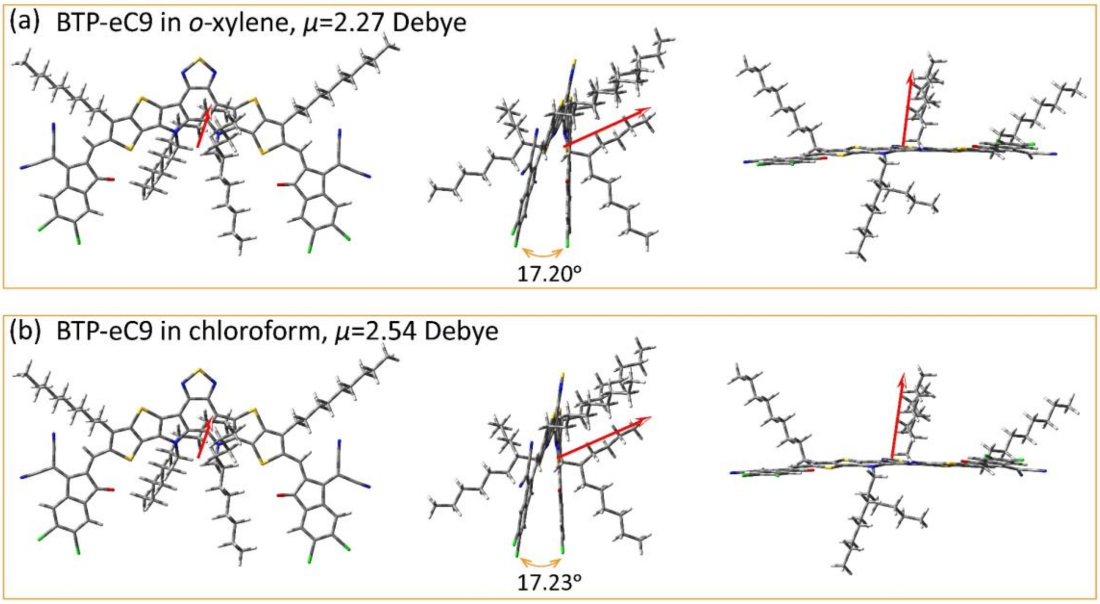

Figure S14 │ Calculated molecular dipole moment. Molecular dipole moments calculated from DFT taking into account **a**, *o*-xylene and **b**, chloroform solvation effects obtained by Gaussian 09 performed at the B3LYP/6-311G(d,p) level.

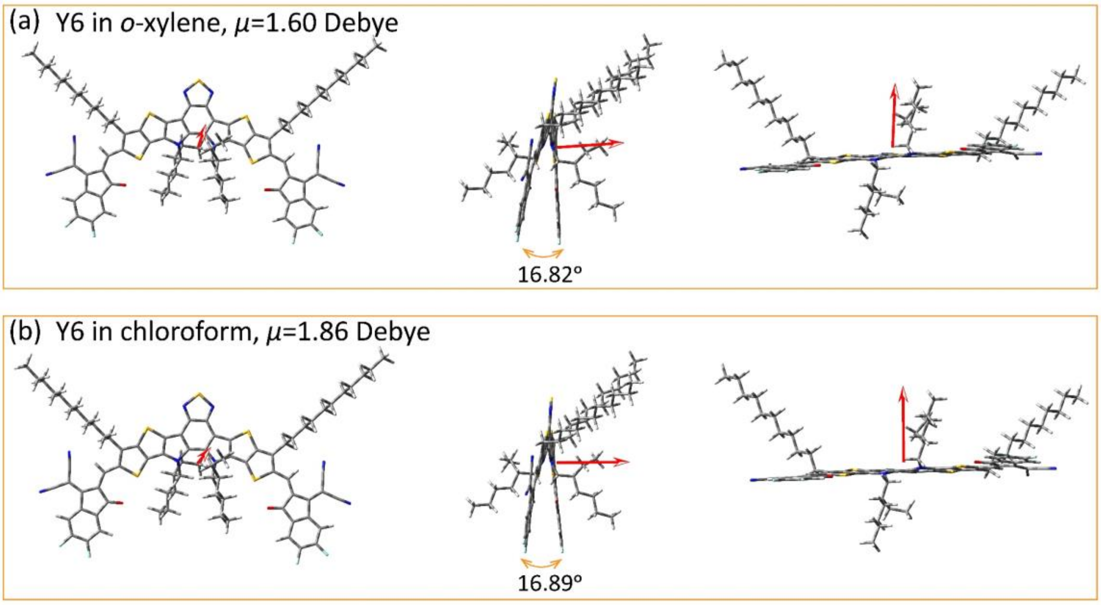

Figure S15 │ Calculated molecular dipole moment. Molecular dipole moments calculated from DFT taking into account **a**, *o*-xylene and **b**, chloroform solvation effects obtained by Gaussian 09 performed at the B3LYP/6-311G(d,p) level.

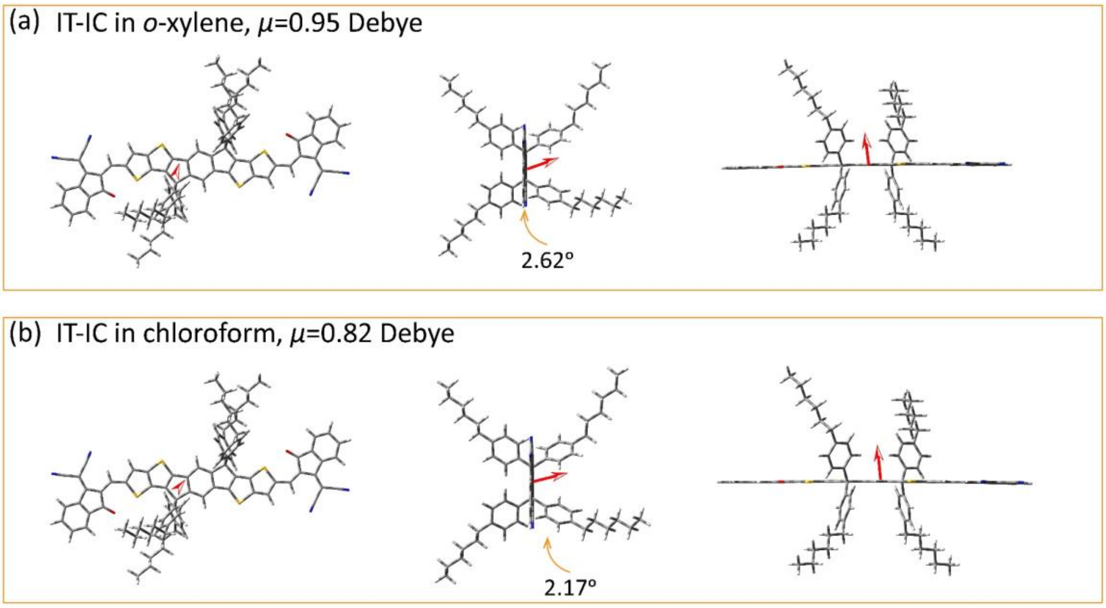

Figure S16 │ Calculated molecular dipole moment. Molecular dipole moments calculated from DFT taking into account **a**, *o*-xylene and **b**, chloroform solvation effects obtained by Gaussian 09 performed at the B3LYP/6-311G(d,p) level.

Supplementary text S5:

Firstly, we applied EEF-coating to devices with CF as processing solvent and inverted structures to explore the compatibility of this strategy. In the case of organic solar cells prepared using CF, a modest increase in PCEs was observed, moving from 15.7% to 16.3% under EEF regulation. More notably, the improvement in inverted devices was even more remarkable, with PCEs increasing from 15.4% to 16.7%. This enhancement can be primarily attributed to the differing polarities of CF and *o*-xy, resulting in PM6 and BTP-eC9 molecules experiencing distinct forces in response to the electric field as will be explained in more detail in subsequent sections.

Absorption spectroscopy employed to investigate changes in photon harvesting capabilities due to differences in molecular packing in the active layer. As shown in **Fig. S13**, the ultraviolet-visible absorption spectra of PM6 and BTP-eC9 films exhibit red-shifted 0-0 transition peaks and increased 0-0/0-1 intensity ratio under the modulation of EEF. These observed alterations, including the red-shifted transition peaks and the modified ratio of J-type aggregation absorption, are indicative of partially more ordered microstructures within the films. These results are consistent with those we obtained in *o*-xylene processed films. The EEF influence on the molecular dipole effectively induces optimized molecular orientation in the transition state and promotes partially ordered microstructures in the film, thereby enhancing the films absorption coefficient.

In this part of the calculation, we use the “SCRF” keyword to establish the implicit solvent environments, which causes the solute to be placed in a cavity within the solvent reaction field to perform the calculation. Instead of specifically describing the exact

structure and distribution of solvent molecules in the vicinity of the solute, the process considers the solvent environment simply as a polarizable continuous medium. Solvation effects can be divided into polar and non-polar components. The polar part reflects the electrostatic interactions between the solvent-solute and also includes the polarization of the solvent on the electron distribution of the solute. Here in the calculations, we have used the dielectric constants provided by the Gaussian: *o*-Xylene: ε=2.5454; Chloroform: ε=4.7113 (Similar to related results described in later).

Calculations indicate that solvents of different polarities have an effect on the dipole moment of non-fullerene acceptor molecules (**Fig. S14 to S16**). For the BTP-eC9 molecule, the highly polar solvent CF decreases the dihedral angle of its malonitrile groups. The improved molecular planarity further leads to a larger molecular dipole moment. The bond angles between the alkyl side chain and the main chain are 1.46° (in *o*-xy) and 1.87° (in CF), respectively. We considered the entire alkyl side-chain structure in our calculations (which is the reason why the molecular dipole moment is nearly parallel to its stacking direction), and the change in the bond angle between it and the main chain is the main factor that leads to the change in its molecular planarity and dipole moment.

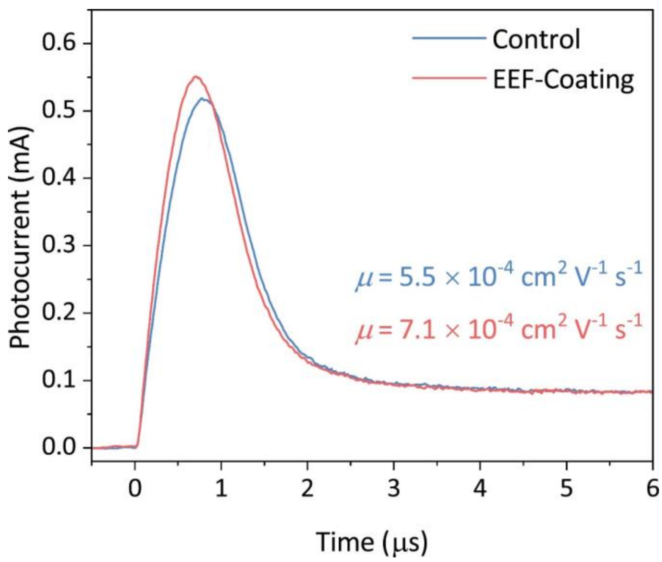

Figure S17 │ Electrical characteristics of charge carriers. The photo-induced charge carrier extraction by linearly increasing voltage curves of the corresponding devices (*o*-xy).

Table S4 │ Parameters obtained from photo-CELIV measurement.

Samples		t_{max} (μs)	j_{max} (mA cm^{-2})	j_0 (mA cm^{-2})
PM6:BTP-eC9	Control	0.67	8.76	1.30
	EEF-Coating	0.81	8.19	1.42

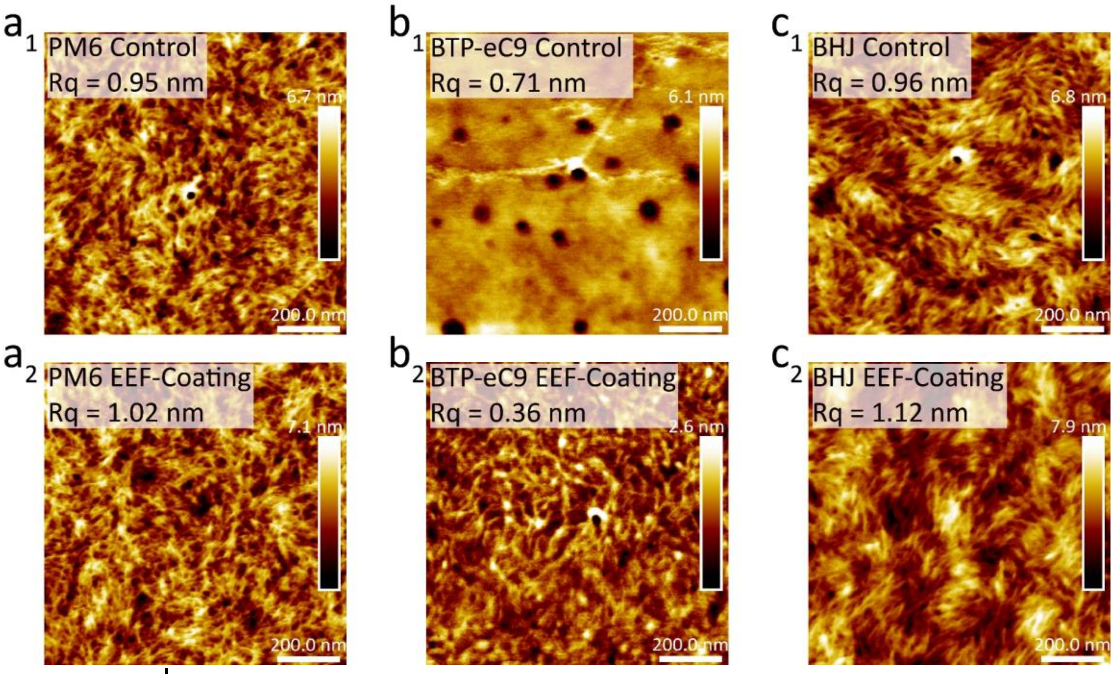

Figure S18 │ Atomic force microscope patterns. The AFM height map for the control and EEF-coated **a**, PM6 (*o*-xy), **b**, BTP-eC9 (*o*-xy), and **c**, PM6:BTP-eC9 (*o*-xy) films.

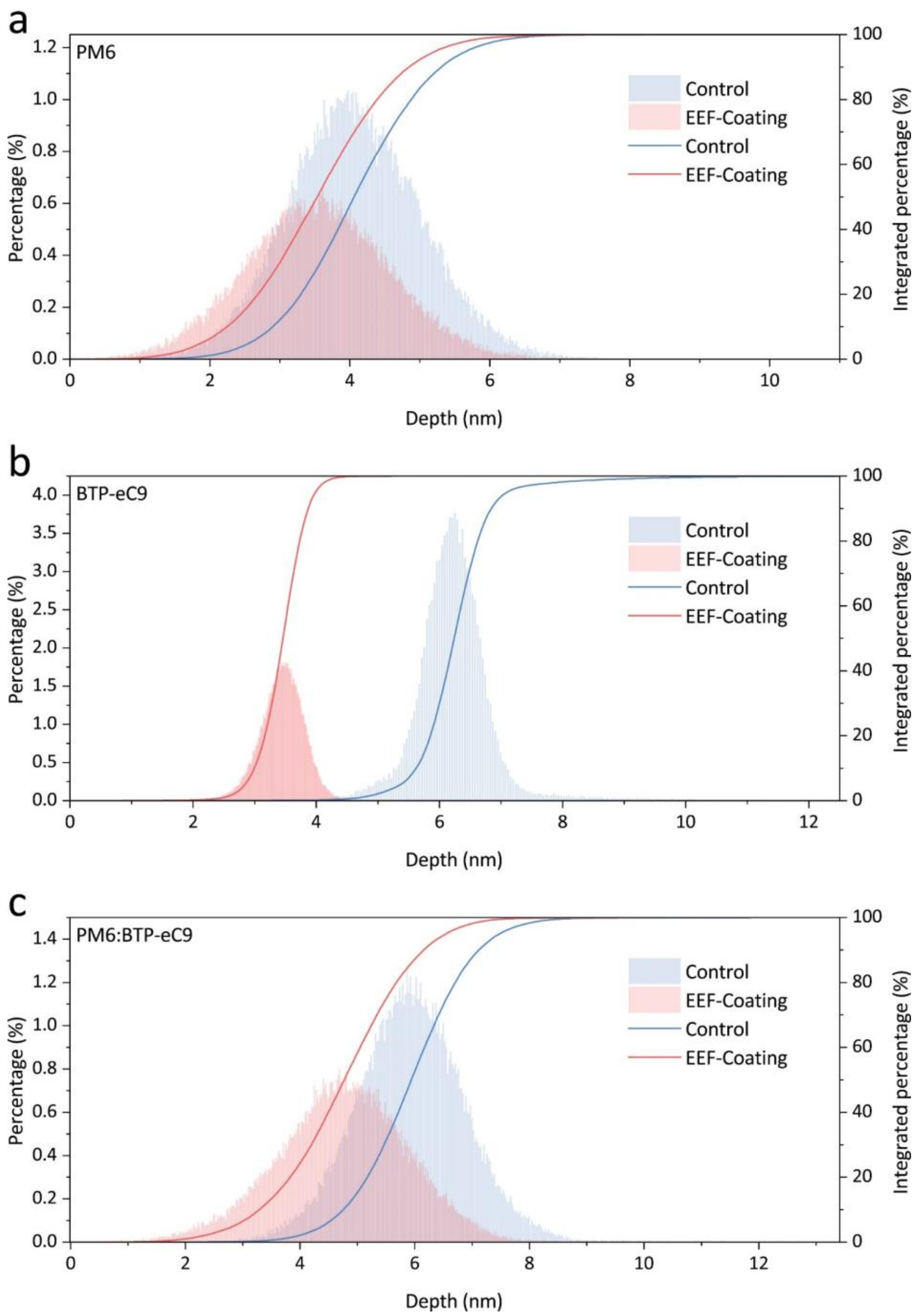

Figure S19 │ Atomic force microscope analysis of thin films. Surface depth features and bearing analysis of AFM image of **a**, PM6 (*o*-xy), **b**, BTP-eC9 (*o*-xy), and **c**, PM6:BTP-eC9 (*o*-xy).

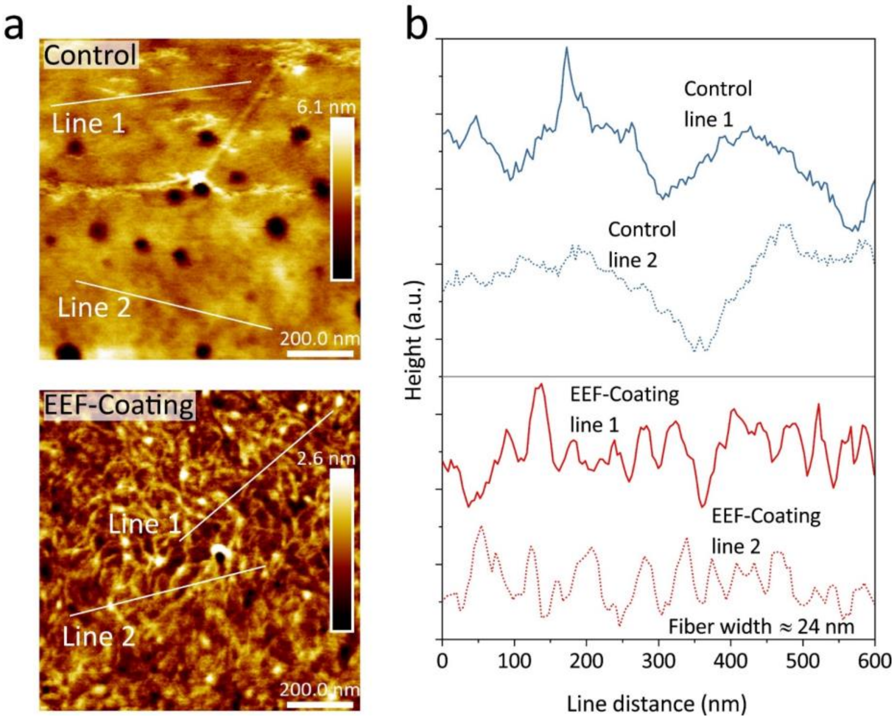

Figure S20 │ Atomic force microscope patterns of thin films. a, AFM height map and **b**, line profiles along the white lines to obtain the fibril morphology for the EEF-coated BTP-eC9 (*o*-xy) film. The fibril width is obtained from the full-width at half-maximum.

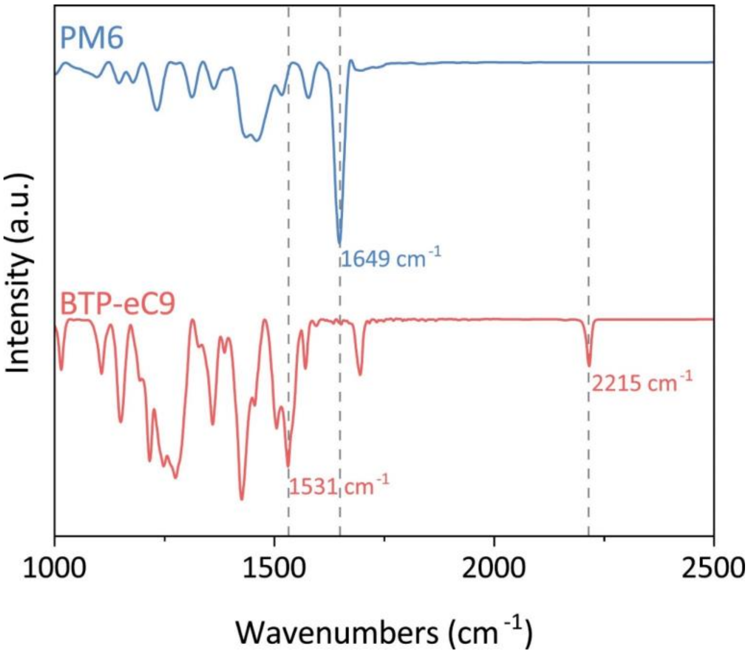

Figure S21 │ Fourier transform infrared spectra. FTIR of PM6 and BTP-eC9, PM6 show a unique peak at 1649 cm^{-1} from alkene vibrations, BTP-eC9 show a unique peak at 1531 cm^{-1} from N-H stretching.

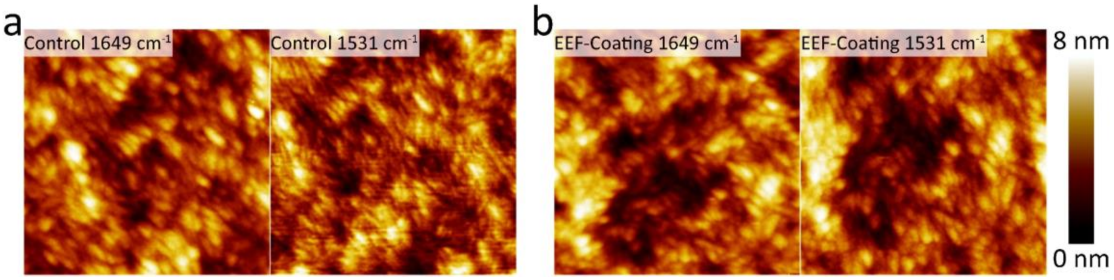

Figure S22 │ AFM height maps. a, Control and **b**, EEF-coated AFM images of PM6:BTP-eC9 blended films obtained during photoinduced force microscopy measuring at corresponding wavenumbers.

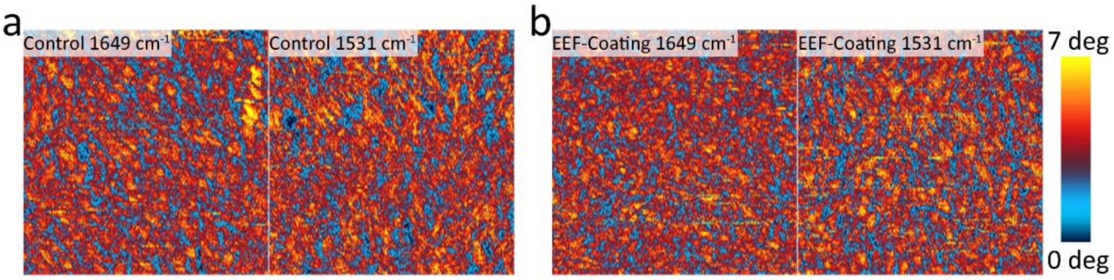

Figure S23 │ AFM phase images. a, Control and **b**, EEF-coated AFM phase images of PM6:BTP-eC9 blended films obtained during photoinduced force microscopy measuring at corresponding wavenumbers.

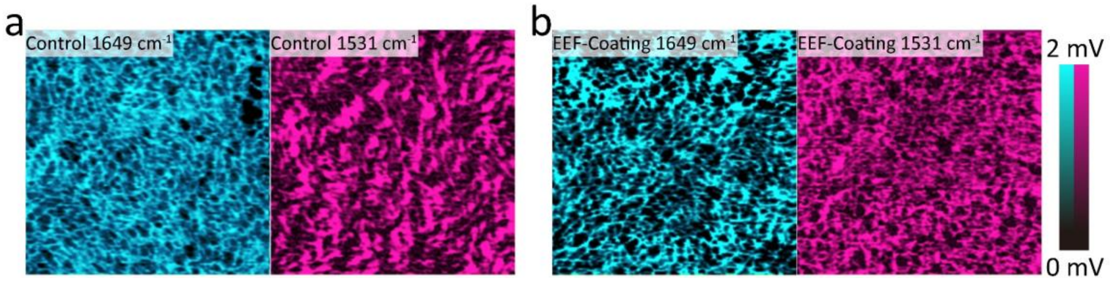

Figure S24 │ PiFM image. a, Control and **b**, EEF-coated photo-induced force microscopy images at a wavenumber of 1,531 cm^{-1} (representing BTP-eC9) and 1,649 cm^{-1} (representing PM6).

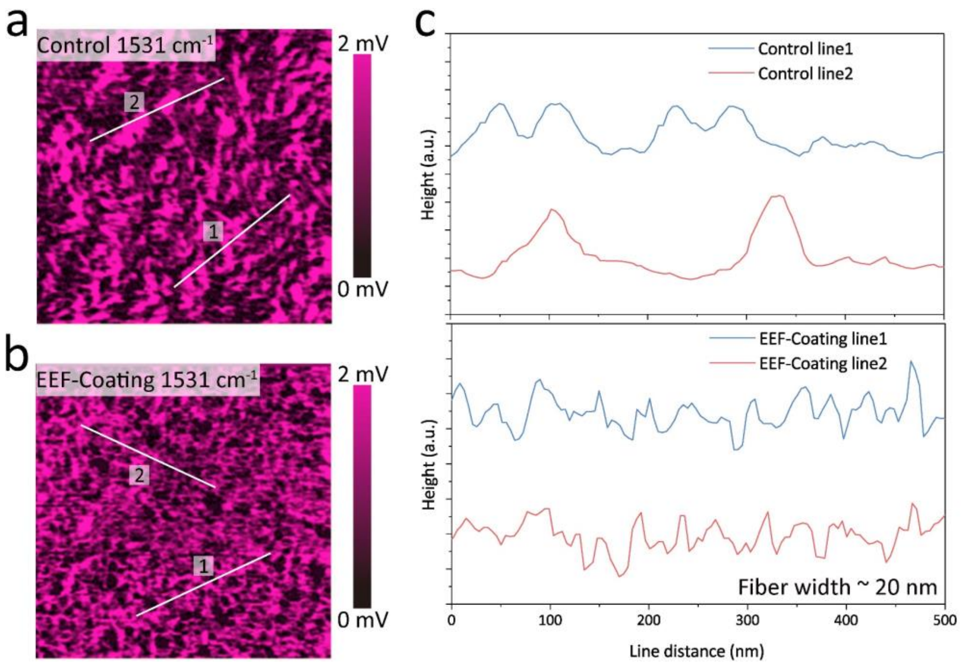

Figure S25 │ PiFM image. a, Control and **b**, EEF-coated photo-induced force microscopy images at a wave number of 1,531 cm^{-1} (representing BTP-eC9) and **c**, the line profiles along the white lines to obtain the fibril width for the PM6:BTP-eC9 (*o*-xy) blended film. The fibril width is obtained from the full-width at half-maximum.

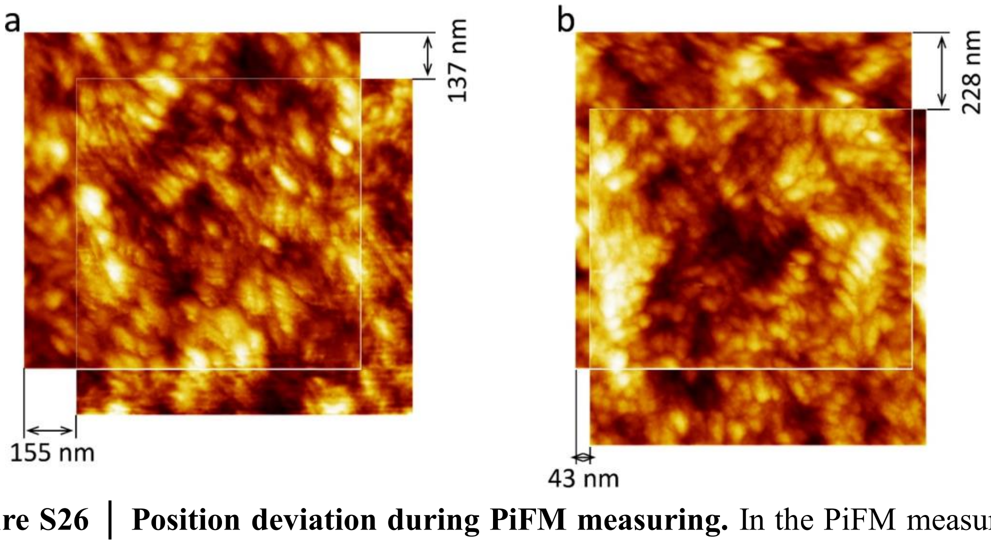

Figure S26 │ Position deviation during PiFM measuring. In the PiFM measuring, there is a relatively small position deviation in the images scanned by two different wavenumbers. This deviation of **a**, control and **b**, EEF-coated films is obtained by comparing the AFM height images.

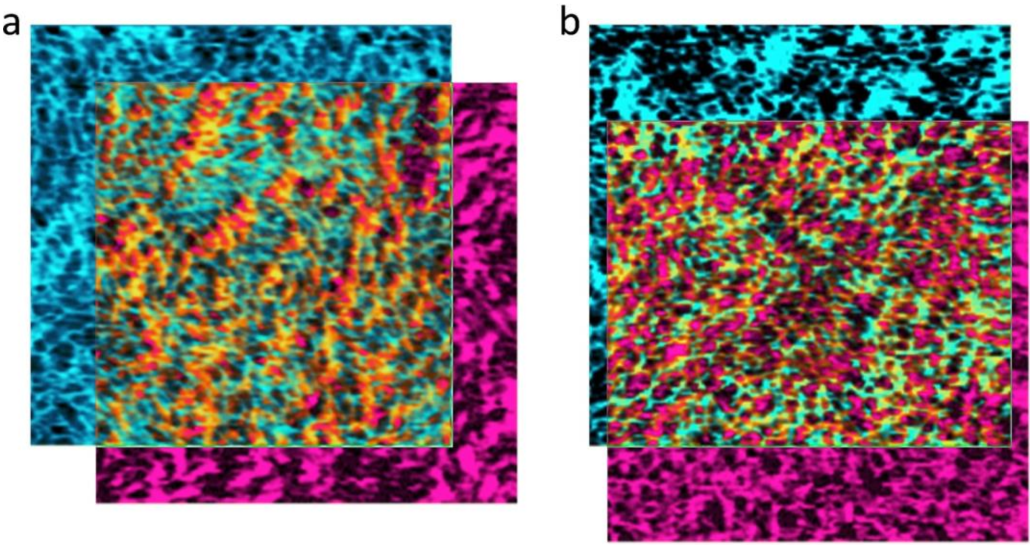

Figure S27 │ Superposition of donor and acceptor PiFM after positional deviation correction. The integration of the **a**, control and **b**, EEF-coated films PiFM images of the two wavenumbers (red 1531 cm^{-1} represents BTP-eC9, green 1649 cm^{-1} represents PM6) after position deviation correction can segment different areas and perform corresponding percentage calculations.

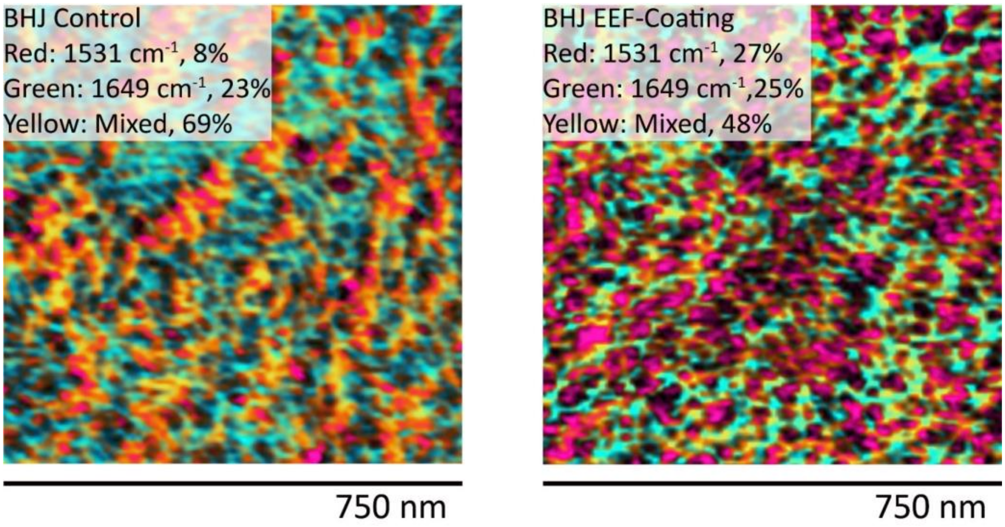

Figure S28 │ Integrated PiFM images. The marked percentages are the proportions of pure domains and mixed domains obtained by segmenting different areas and performing corresponding calculations.

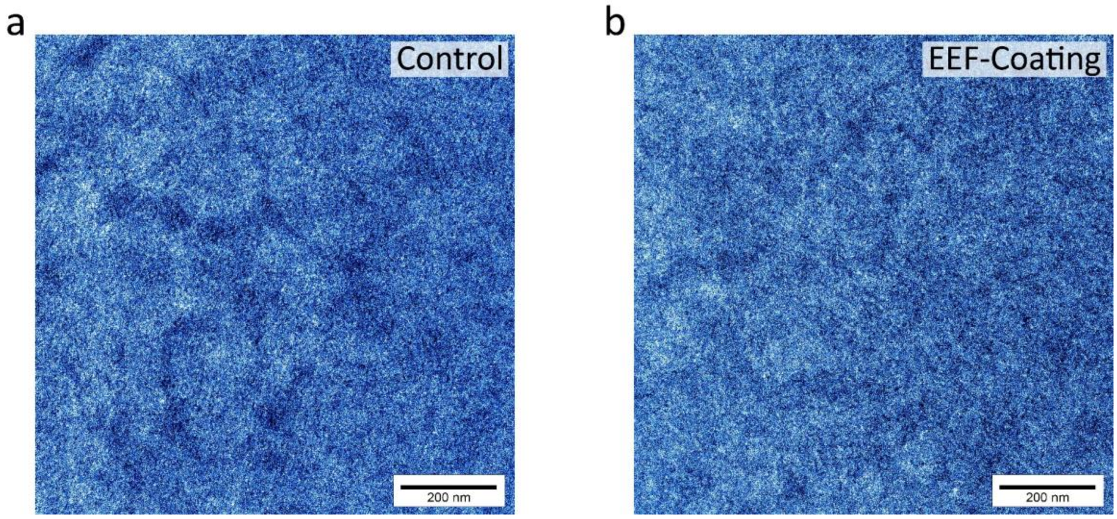

Figure S29 │ Transmission electron microscope images. TEM images of **a**, control and **b**, EEF-coated PM6:BTP-eC9 (*o*-xy) films.

Supplementary text S6:

Photo-induced force microscopy (PiFM) is a scan probe technique that offers images with spectroscopic contrast at a spatial resolution in the nanometer range(*2*). It combines the principles of atomic force microscopy (AFM) with infrared (IR) spectroscopy to achieve high-resolution chemical mapping and characterization of materials at the nanoscale. PiFM is based on the interaction between a sharp AFM tip and the photothermal effect induced by infrared light absorption in a sample. The working principle of PiFM can be divided into three main steps: a) Infrared Illumination: The IR laser is focused on the AFM tip-sample junction. When the sample absorbs IR radiation, it undergoes localized heating, leading to a temporary thermal expansion. b) Tip-Induced Photothermal Response: As the sample undergoes thermal expansion, the AFM tip, in close proximity, experiences a change in its contact force due to the sample's thermal expansion. This change in force is a result of the photothermal interaction. c) Detection: The AFM system records the deflection of the AFM tip, which

is proportional to the photothermal response induced by the IR absorption. This deflection signal is then used to create high-resolution chemical maps and spectroscopic data.

According to Fourier transform infrared spectroscopy (**Fig. S21**), PM6 show a unique peak at a wavenumber of 1649 cm^{-1} from alkene vibrations, BTP-eC9 show a unique peak at a wavenumber 1531 cm^{-1} from N-H stretching(*3*). The absorption at these two different wave numbers provides a window for us to use PiFM to distinguish the crystal structure of the donor and acceptor in the active layer at the nanometer level. During the PiFM scanning, we employed 1649 cm^{-1} and 1531 cm^{-1} IR lasers in the same region of active layers to obtain the distribution and structure of the donor and NFA, respectively. As we see in **Fig. S24**, the shape of PM6 obtained at 1649 cm^{-1} IR laser exhibits an approximate fiber-like structure in both control and EEF-coated blends. While the shapes of the control and EEF-coated NFA obtained under the irradiation of an IR laser at 1531 cm^{-1} exhibited very large differences in the blends. Induced by the external electric field, the NFA in the EEF-coated blends exhibited a fiber-like structure with a diameter of about 20 nm acting like a polymer (**Fig. S25**). This structure indicates that the fiber-like structure induced by the electric field in neat BTP-eC9 is maintained in the blended films. And it is identical to the results we got from our analysis in GIWAXS.

However, some interruptions were encountered when we were preparing to integrate the PiFM images obtained under IR laser irradiation at 1649 cm^{-1} (represents PM6) and 1531 cm^{-1} (represents BTP-eC9). There was a slight positional drift of the

substrate relative to the probe due to switching between different wavelengths of the IR laser during our performed PiFM scans. Therefore, the AFM height images obtained during two scans based on infrared lasers of different wavelengths were used to correct for this positional drift (**Fig. S26**). After correcting for this positional drift, PM6 (images obtained under 1649 cm^{-1} laser irradiation) and BTP-eC9 (images obtained under 1531 cm^{-1} laser irradiation) were integrated to obtain the true morphology of the active layer. **Fig. S27** presents the integrated PiFM images, according to which the different regions can be segmented and the corresponding percentages calculated. The size of the original PiFM image at different wave numbers was 1 μm × 1 μm, and the integrated image size was reduced due to positional drift correction. Therefore, we took 750 nm × 750 nm (**Fig. S28**) on each of the integrated PiFM images in the control and EEF-coated blends for analysis. According to the segmentation calculation, the ratio of PM6:BTP-eC9:mixed domains in the control blend is 23%:8%:69%, while the value of this ratio in the EEF-coated blend is 25%:27%:48%.

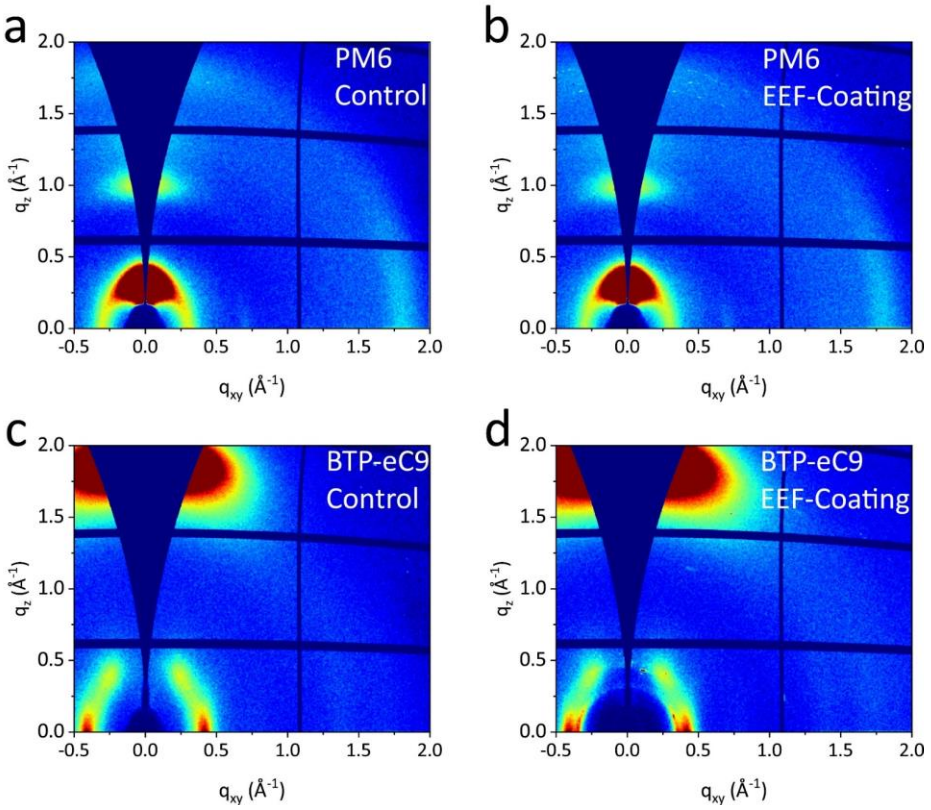

Figure S30 │ 2D GIWAXS measurements. 2D GIWAXS patterns of **a**, PM6 (*o*-xy), **b**, EEF-coated PM6 (*o*-xy), **c**, BTP-eC9 (*o*-xy), and **d**, EEF-coated BTP-eC9 (*o*-xy).

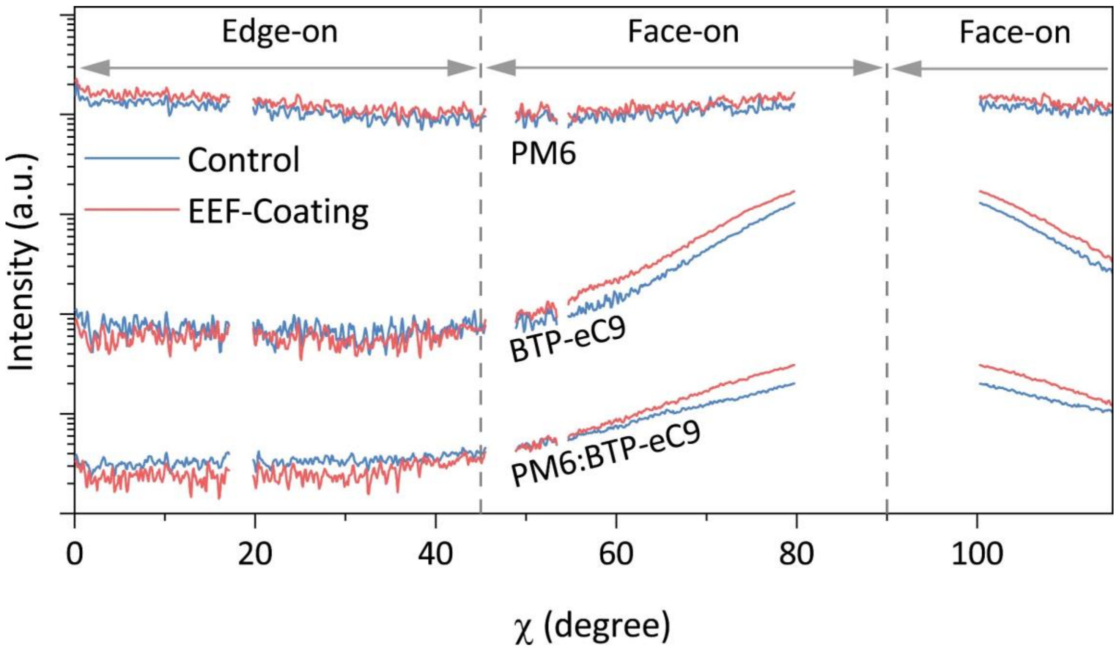

Figure S31 │ Integrated scattering profiles. Pole figures extracted from the (010) diffractions for corresponding films. The integrated area of the polar angle χ in the range 0-45° (0--45°) and 45-90° (-45--90°) is defined as the ratio of face-on (light blue area) and edge-on (light pink area) crystallites, respectively.

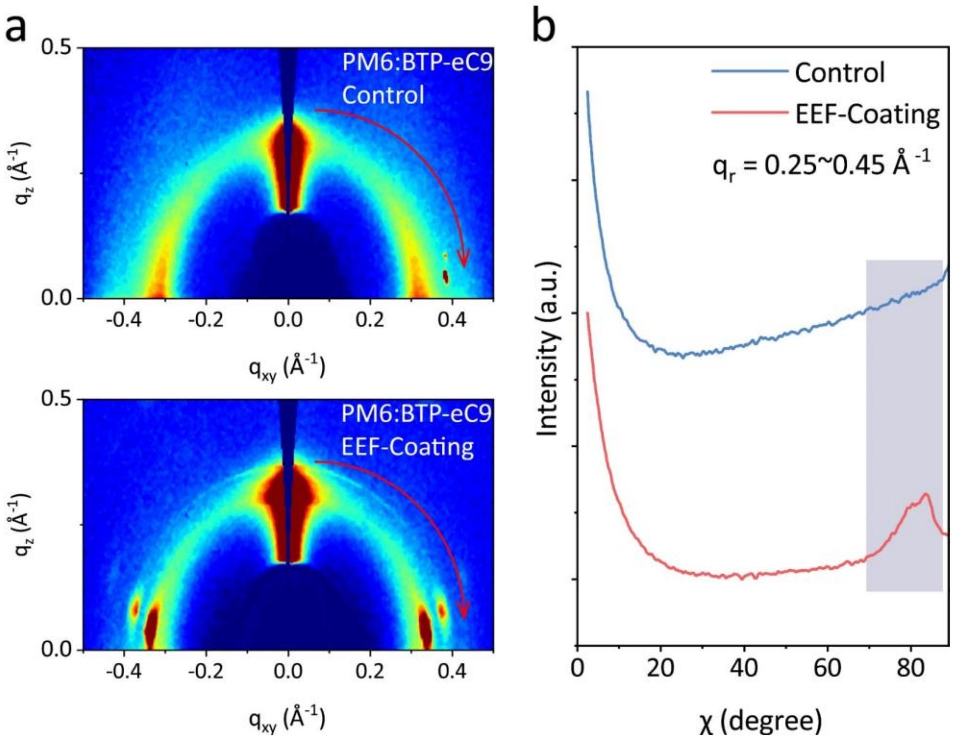

Figure S32 │ Integrated scattering profiles. a, 2D GIWAXS patterns and **b**, pole figures extracted from the (100) diffractions for corresponding films (*o*-xy).

Table S5 │ Lamellar peak (100) position of PM6 (*o*-xy) films with corresponding d-spacing and CCL in IP direction.

Samples		PM6 Lamellar peak (in-plane)			
		Peak (Å^{-1})	d-spacing (Å)	FWHM (Å^{-1})	CCL (Å)
PM6	Control	0.30	20.94	0.09	62.83
	EEF-Coating	0.31	20.26	0.09	62.83
PM6:BTP-eC9	Control	0.31	20.27	0.06	94.24
	EEF-Coating	0.32	19.63	0.07	80.78

Table S6 │ π-π peak (010) position of PM6 (*o*-xy) films with corresponding d-spacing and CCL in OOP direction.

Samples		PM6 π-π peak (out-of-plane)			
		Peak (Å^{-1})	d-spacing (Å)	FWHM (Å^{-1})	CCL (Å)
PM6	Control	1.78	3.53	0.19	29.76
	EEF-Coating	1.80	3.50	0.17	33.26

Table S7 │ The ratio of face-on (0-45°) and edge-on (45-90°) orientation for the films.

Samples		Face-on (%)	Edge-on (%)	Face-on/Edge-on
PM6	Control	41.68	58.32	0.71
	EEF-Coating	42.28	57.72	0.73
BTP-eC9	Control	78.36	21.64	3.62
	EEF-Coating	86.04	13.96	6.16
PM6:BTP-eC9	Control	68.51	31.49	2.18
	EEF-Coating	78.90	21.10	3.74

Table S8 │ Lamellar peak (100) and (200) position of BTP-eC9 in BTP-eC9 (*o*-xy) and PM6:BTP-eC9 (*o*-xy) films with corresponding d-spacing and CCL in IP direction.

Samples		BTP-eC9 Lamellar peak A (in-plane)				BTP-eC9 Lamellar peak B (in-plane)			
		Peak ($Å^{-1}$)	d-spacing (Å)	FWHM ($Å^{-1}$)	CCL (Å)	Peak ($Å^{-1}$)	d-spacing (Å)	FWHM ($Å^{-1}$)	CCL (Å)
BTP-eC9	Control	0.41	15.32	0.13	43.50	/	/	/	/
	EEF-Coating	0.41	15.32	0.12	47.12	0.34	18.48	0.03	188.50
PM6:	Control	0.38	16.53	0.20	28.27	/	/	/	/
BTP-eC9	EEF-Coating	0.40	15.71	0.13	43.50	0.34	18.48	0.04	141.37

Table S9 │ π-π stacking peaks (010) position of BTP-eC9 in BTP-eC9 (*o*-xy) and PM6:BTP-eC9 (*o*-xy) films with corresponding d-spacing and CCL in OOP direction.

Samples		BTP-eC9 π-π peak A (in-plane)				BTP-eC9 π-π peak B (in-plane)			
		Peak ($Å^{-1}$)	d-spacing (Å)	FWHM ($Å^{-1}$)	CCL (Å)	Peak ($Å^{-1}$)	d-spacing (Å)	FWHM ($Å^{-1}$)	CCL (Å)
BTP-eC9	Control	1.87	3.36	0.37	15.28	/	/	/	/
	EEF-Coating	1.86	3.38	0.36	15.71	/	/	/	/
PM6:	Control	1.85	3.40	0.34	16.63	/	/	/	/
BTP-eC9	EEF-Coating	1.84	3.41	0.32	17.67	0.60	10.47	0.09	62.83

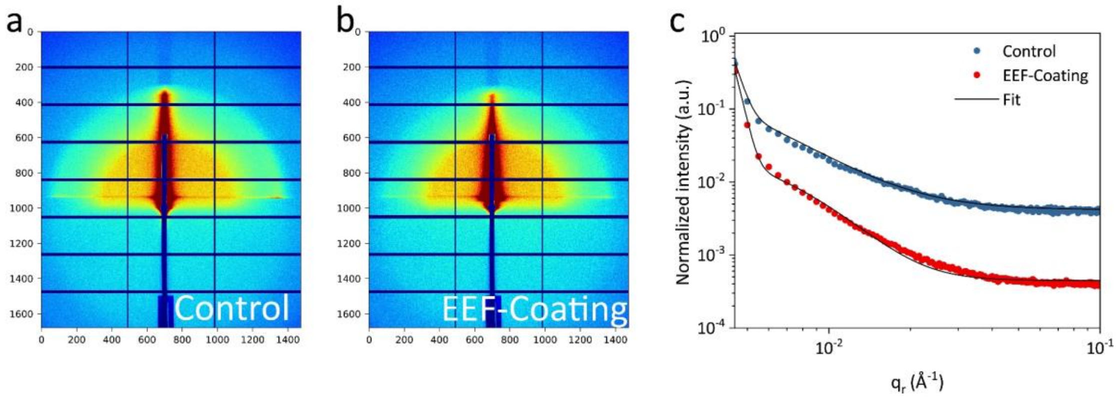

Figure S33 │ 2D GISAXS measurements. 2D GISAXS patterns of **a**, PM6:BTP-eC9 (*o*-xy) and **b**, EEF-coated PM6:BTP-eC9 (*o*-xy). **c**, Corresponding fitting results.

Table S10 │ Morphology parameters fitted by the GISAXS profiles (ξ is the intermixing domain size; 2Rg is the crystal domain size).

**Samples**		**ξ (nm)**	**2Rg (nm)**
PM6:BTP-eC9	Control	28.37	20.04
	EEF-Coating	9.07	21.12

Supplementary text S7:

Mixed domain dimension reduction in PiFM images, which is similar to our conclusions obtained in GISAXS, will effectively inhibit bimolecular recombination thus correlating with the filling factor and short-circuit current of OSCs. Acceptor domains occupy an increased proportion and form a continuously entangled 3D nanofiber packing network with donor fibers, which contributes to exciton dissociation and suppresses bimolecular recombination during free electron transport.

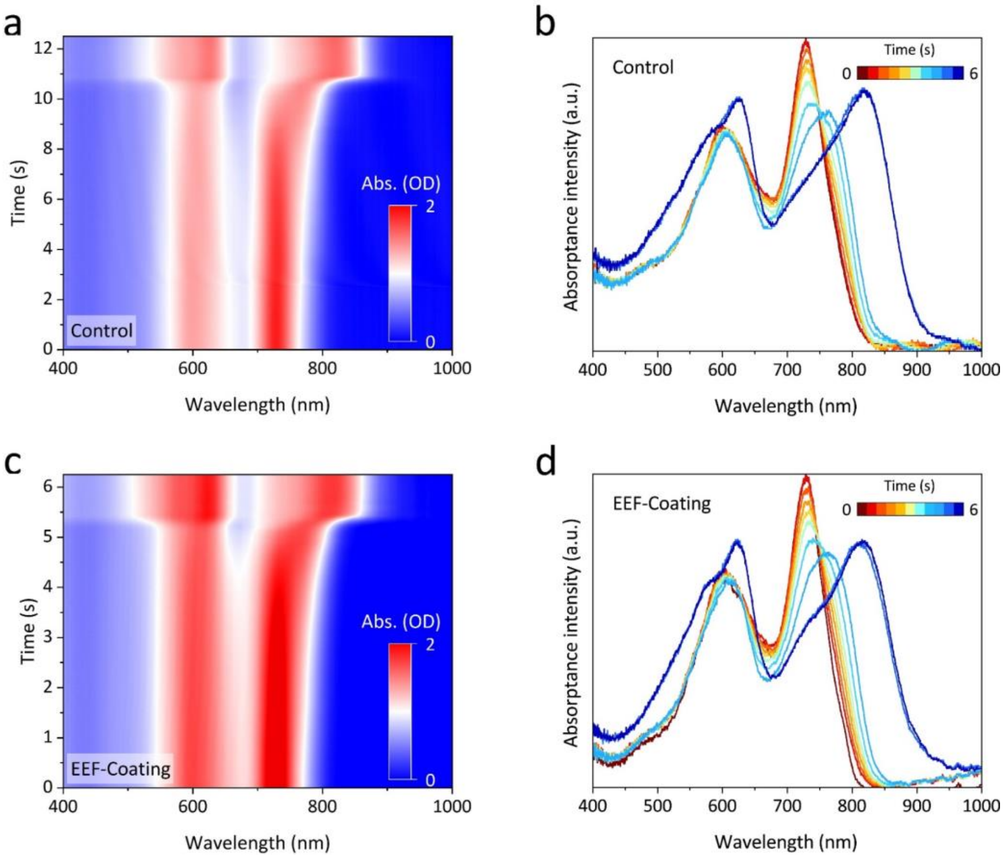

Figure S34 │ In-situ UV-vis absorption. a, **b**, Time-dependent contour maps of UV-vis absorption spectra and corresponding 1D spectra for control PM6:BTP-eC9 during traditional blade coating. **c**, **d**, Time-dependent contour maps of UV-vis absorption spectra and corresponding 1D spectra for PM6:BTP-eC9 during EEF-coating.

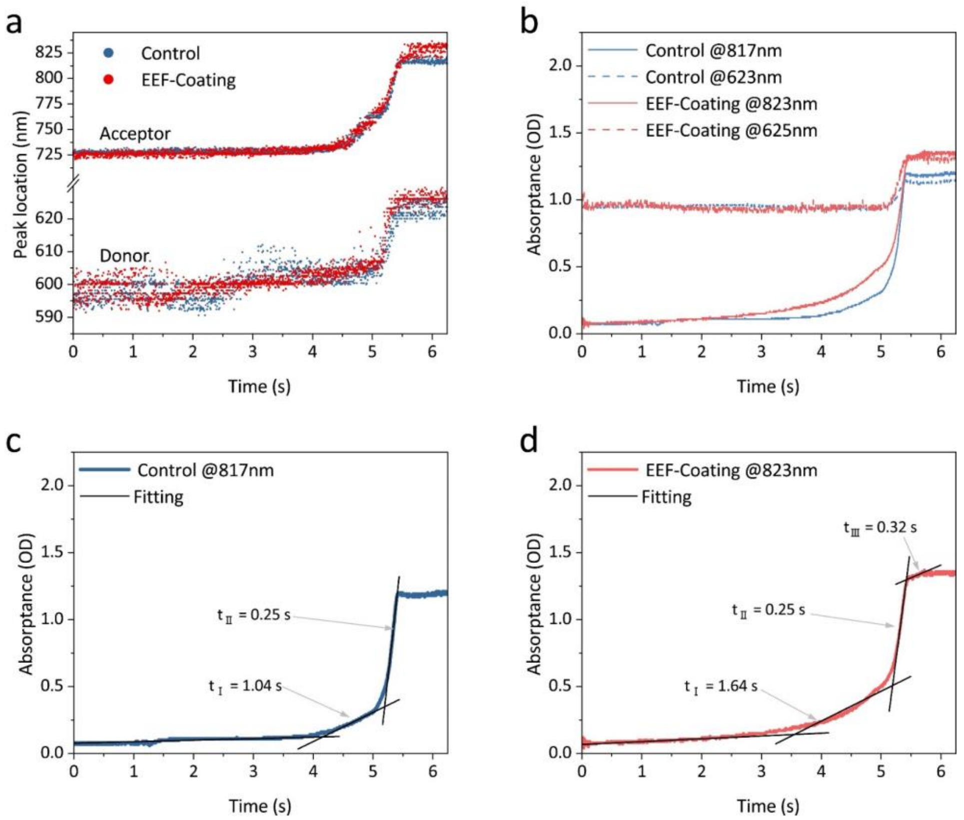

Figure S35 │ Extraction of the crystallization process. a, Time evolution of peak location of PM6 and BTP-eC9 in blends during blade coating. **b**, Time evolution of peak intensity of PM6 and BTP-eC9 in blends during blade coating. **c**, Controlled blend has crystallization process includes nucleation (2.0 s) and crystal growth (0.5 s). **d**, EEF-processed blend crystallization process includes EEF-modulated nucleation (4.0 s), crystal growth (0.5 s), and fine-tuning (0.6 s) stages.

Supplementary text S8:

The variation of in situ absorption intensity is shown in **Fig. S35**. Depending on the aggregation behavior of acceptor materials during film formation, several distinct stages can be defined in the evolution of absorbance. (I) Rapid solvent evaporation stage: The solvent (*o*-xy) exhibits rapid evaporation while the molecules approach each other and start to exhibit pre-aggregation (crystal nucleation) behavior. (II) Rapid crystal growth stage: self-assembly of molecules, and the resulting crystal nucleus grows gradually. (III) Fine-tuning of the film-forming stage: The hybrid film shows

further densification under the action of an external electric field (this stage is exclusive to EEF-coated films). Alternatively, changes in the intensity of in situ absorption due to changes in evaporation rate can be ruled out. When there is a significant difference in the rate of solvent evaporation, we will observe a clear difference in the change in peak position, which is not actually the case. The reason for the change in the in-situ absorption intensity is due to the dielectrophoretic forces (as discussed later) changing the concentration of donor or acceptor molecules in the wet film at different height positions from the substrate. As discussed later, dielectrophoretic forces push the acceptor molecules towards the surface of the wet film (in *o*-xylene), resulting in a larger concentration of acceptor molecules at the surface, reaching the concentration threshold for the formation of crystallization sites ahead of time. However, the evaporation of the solvent during this process is still the main process affecting the crystallization of molecules in the kinetics of film formation.

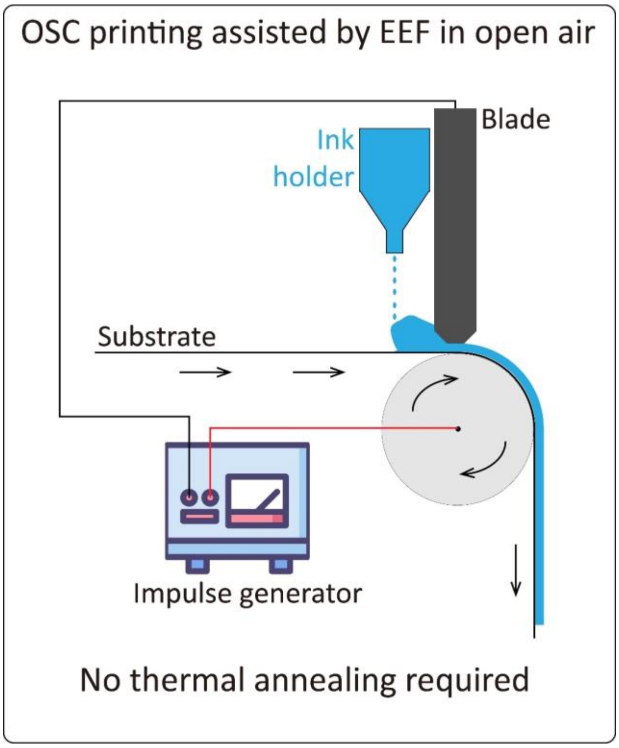

Figure S36 │ Schematic illustration of EEF assisted fab production of OSCs. The envisioned blue-print of annealing-free OSCs industrial printing assisted by EEF in open-air.

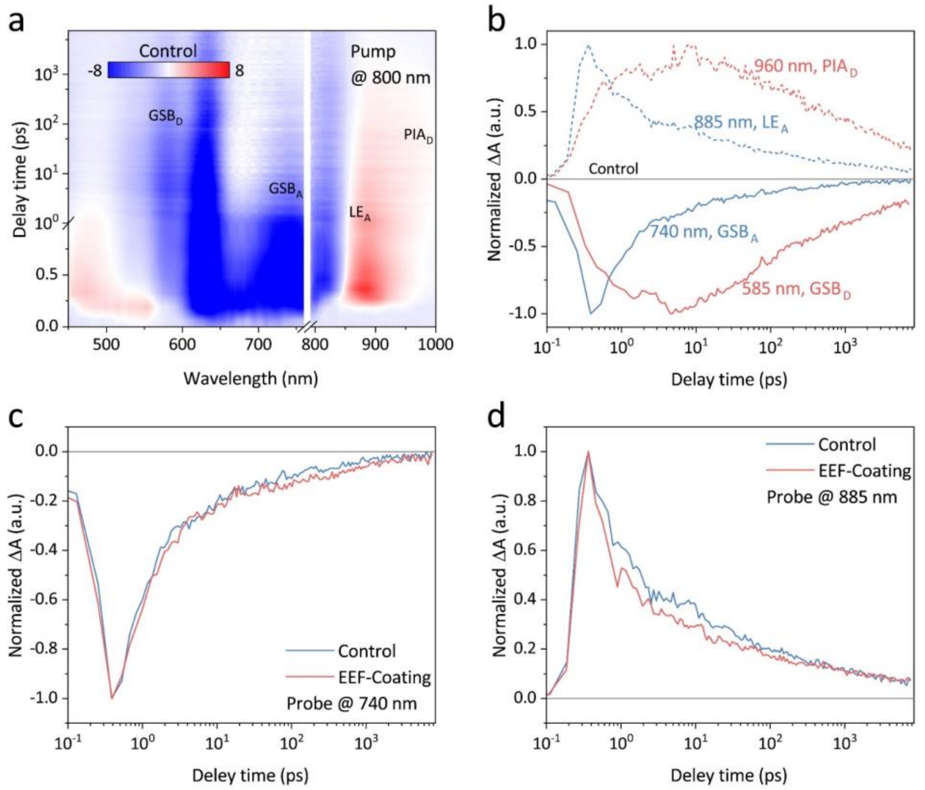

Figure S37 │ Charge dynamics. a, 2D TA data recorded from the control PM6:BTP-eC9 (*o*-xy) blend excited by 800 nm pulsed beam. **b**, Decay dynamics probed at 585 nm, 740 nm, and 885 nm from the controlled PM6:BTP-eC9 (*o*-xy) film. **c**, Ground state bleaching of acceptor at 740 nm. **d**, HT signals of acceptor in controlled and EEF-processed PM6:BTP-eC9 (*o*-xy) films.

Table S11 │ Parameters of acceptor localized exciton decay dynamics for PM6:BTP-eC9 probe at 885 nm.

Samples		$\tau_{1,\ 885\ nm}$ (ps)	$A_{1,\ 885\ nm}$ (%)	$\tau_{2,\ 885\ nm}$ (ps)	$A_{2,\ 885\ nm}$ (%)	$\tau_{AVG,\ 885\ nm}$ (ps)
PM6:BTP-eC9	Control	0.89	74.60	82.56	25.40	21.64
	EEF-coating	0.58	83.09	63.64	16.91	11.25

Table S12 │ Parameters of charge transfer and exciton dynamics for PM6:BTP-eC9 probe at 585 nm.

Samples		$\tau_{1,\ 585\ nm}$ (ps)	$A_{1,\ 585\ nm}$ (%)	$\tau_{2,\ 585\ nm}$ (ps)	$A_{2,\ 585\ nm}$ (%)	$\tau_{AVG,\ 585\ nm}$ (ps)	K_{HT} (ps^{-1})
PM6:BTP-eC9	Control	0.17	88.67	1.62	11.32	0.33	3.03
	EEF-coating	0.13	77.60	0.75	22.40	0.27	3.70

Table S13 │ Parameters of donor charge dynamics for PM6:BTP-eC9 probe at 960 nm.

Samples		$\tau_{rise, 960\ nm}$ (ps)	$\tau_{decay, 960\ nm}$ (ps)
PM6:BTP-eC9	Control	0.30	1.42
	EEF-coating	0.16	1.75

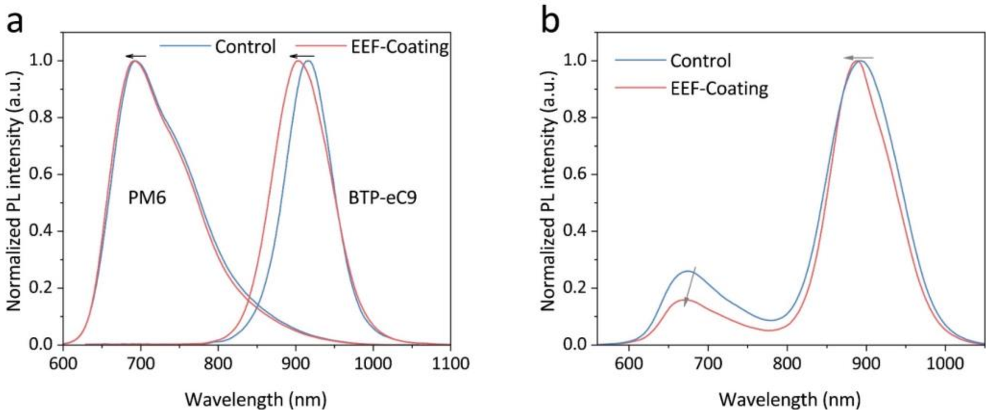

Figure S38 │ Photoluminescence spectra. a, Photoluminescence spectra of PM6 (*o*-xy) and BTP-eC9 (*o*-xy). **b**, Photoluminescence spectra of PM6:BTP-eC9 (*o*-xy) films.

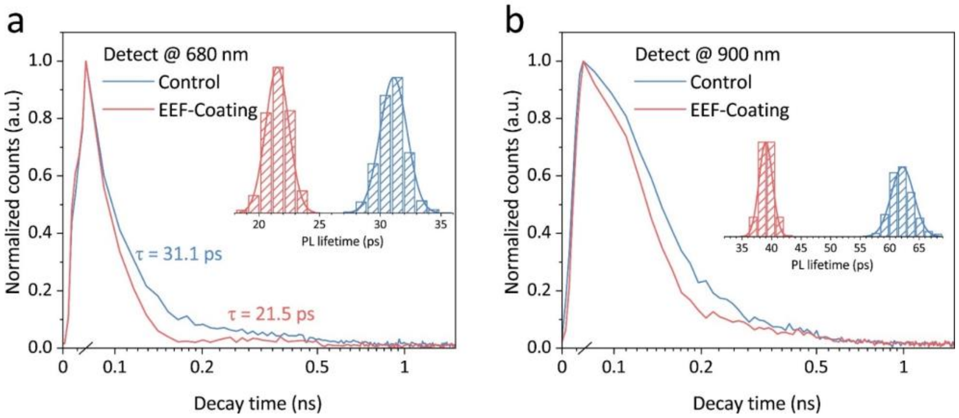

Figure S39 │ Time-resolved photoluminescence. a, TRPL decay excited by 400 nm pulses and probe at 680 nm of PM6 (*o*-xy) PL peak. **b**, TRPL decay excited by 400 nm pulses and probe at 900 nm of BTP-eC9 (*o*-xy) PL peak.

Table S14 │ Time-resolved photoluminescence spectroscopy parameters of exciton dynamics for PM6:BTP-eC9 detected at 900 nm.

Samples		τ_1 (ps)	A_1 (%)	τ_2 (ps)	A_2 (%)
PM6:BTP-eC9	Control	54.6	94.9	215.8	5.1
	EEF-coating	35.6	98.2	251.7	1.8

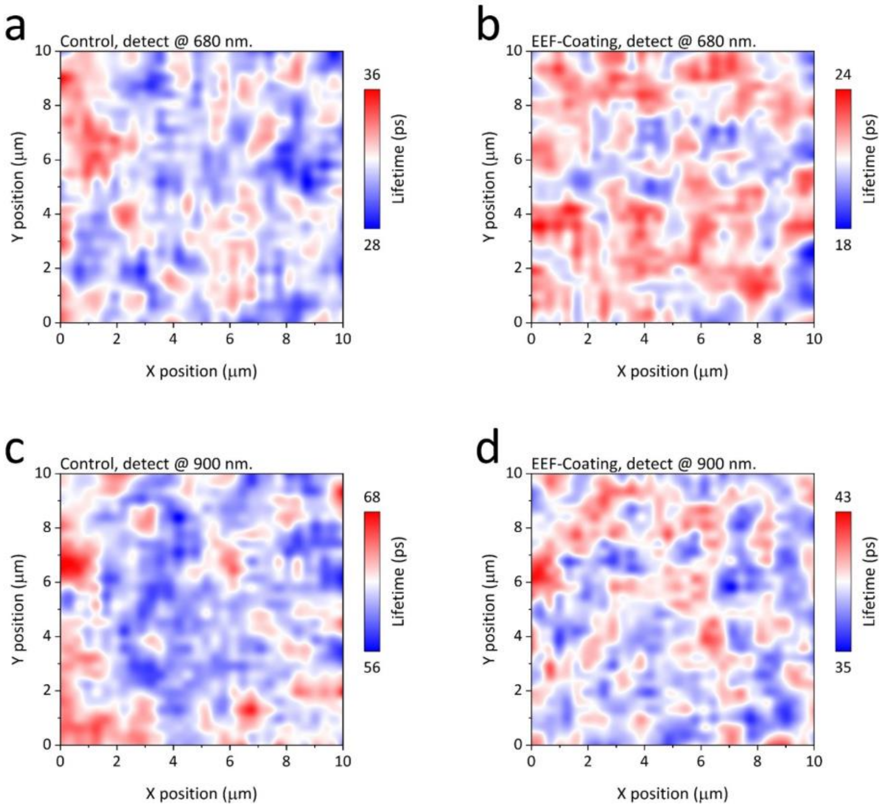

Figure S40 │ Time-resolved photoluminescence mapping. a, **b**, TRPL mapping images of control and EEF-coated PM6:BTP-eC9 (*o*-xy) blended films, respectively, excited by 400 nm pulses and probe at 680 nm of PM6 PL peak. **c**, **d**, TRPL mapping images excited by 400 nm pulses and probe at 900 nm of BTP-eC9 (*o*-xy) PL peak.

Supplementary text S9:

Photoluminescence (PL) spectroscopy was employed to investigate exciton dynamics further. EEF-coated films (*o*-xylene) exhibited a blue-shift in PL peak position, especially in acceptor films compared to control films (**Fig. S38**). This shift, alongside the red-shift in absorption peak position, results in a smaller energy gap

between emitted and absorbed photons. In organic heterojunctions, interfaces between (donor/acceptor) molecules with differing electron affinities commonly occur, and these interfaces may give rise to exciton dissociation and charge recombination phenomena(*30*). Exciton dissociation and charge recombination interfaces are more prevalent within the mixed phase region of the donor-acceptor blend. The presence of a mixed phase is advantageous in providing an ample interface for exciton dissociation; however, an excessively large mixed phase domain scale can result in an undue recombination loss of charge carriers at molecular interfaces. Conversely, a pure phase contributes to the suppression of charge recombination, but an excessively large pure phase scale is unfavorable for exciton diffusion and dissociation. Therefore, the mixed phase typically exhibits a faster exciton quenching lifetime, while a slower exciton quenching lifetime signifies that the region is closer to a pure phase.

Time-resolved photoluminescence (TRPL) of the acceptor in the EEF-coated blend at 900 nm revealed a shorter lifetime of 35.6 ps and a higher ratio of 98.2%, pointing to swift acceptor exciton dissociation (**Fig. S39**). The slower component, linked to undissociated exciton recombination, was notably reduced in the EEF-coated blend (**Table S14**). Donor TRPL in the EEF-coated blend at 680 nm showed a lifetime of 21.5 ps, indicating a faster energy transfer process. TRPL mapping highlighted well-defined fibrillar connections in the pure acceptor phase of the EEF-coated film, absent in the control film (**Fig. S40**), corroborated by AFM and PiFM results. Gaussian statistics of TRPL mapping aligned with TRPL findings (insert in **Fig. S39**). Time-resolved photoluminescence imaging reveals that the acceptors coated by EEF-coating form a

continuous structure within the heterojunction. Moreover, both the mixed domains of donors and acceptors, as well as their respective pure phase distributions, are uniform. Effectively reducing the mixed phase dimensions, while ensuring efficient exciton dissociation, can suppress carrier recombination. In contrast, the heterojunctions in the control group display larger mixed domains of donors and acceptors, exacerbating free carrier recombination within these domains. These observations align with the results obtained from GIWAXS, GISAXS, and transient absorption spectra.

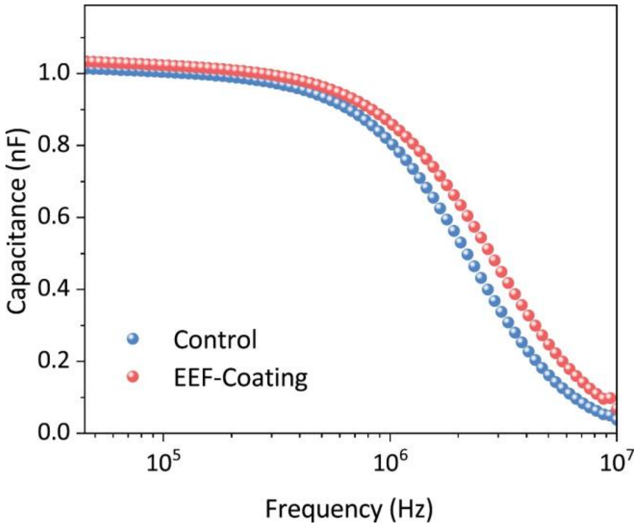

Figure S41 │ Impedance spectra. Capacitance-frequency characteristics of the control and EEF-coated PM6:BTP-eC9 cells.

Supplementary text S10:

The density of states (DoS) can be calculated form capacitance spectroscopy measured in dark environment. The frequency axis can be scaled to energy axis through the follows:

$$E_{\omega} = kT \ln\left(\frac{2\nu_0}{\omega}\right)$$

where ω is the angular frequency calculated by $\omega = 2\pi f$, ν_0 is the attempt-to-escape

frequency of 10^9 Hz. The trap density at energy E_ω can be acquired as:

$$N_t(E_\omega) = -\frac{v_{bi}\, dC\omega}{qd\omega kT}$$

d is the thickness of the active layer and V_{bi} is the built-in voltage measured through Mott-Schottky characterization (**Fig. S41**). Then the energy distribution can be described with Gaussian shape distribution:

$$N_t(E_\omega) = \frac{N_t}{\sqrt{2\pi}\sigma} \exp\left[-\frac{(E_t - E)^2}{2\sigma^2}\right]$$

Where N_t is the total density, E_t is the center of the DoS, σ is the disorder parameter.

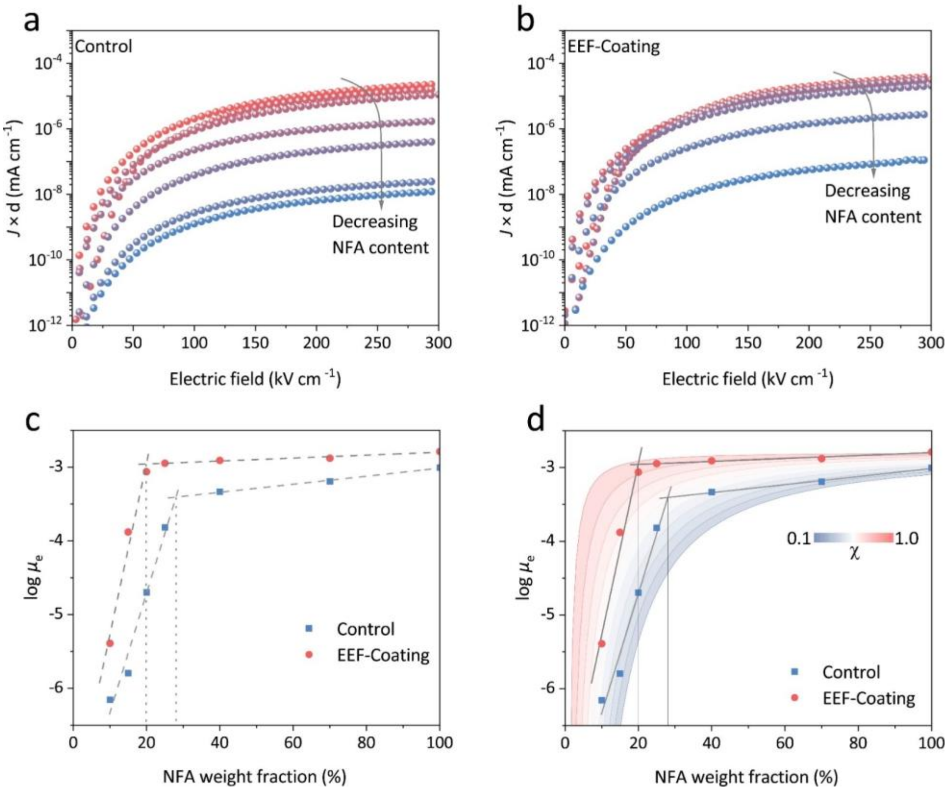

Figure S42 │ Electron mobilities of BTP-eC9 films diluted by PM6 in different ratios. a, **b**, Electron current density × film thickness of devices as a function of the applied electric field for control and EEF-coated PM6:BTP-eC9 with various NFA fractions**. c**, **d**, Logarithm of electron mobilities as a function of acceptor weight fraction for control PM6:BTP-eC9 (blue squares) and EEF-coated PM6:BTP-eC9 (red circles). The unit of μ_e is cm^2 V^{-1} s^{-1}, and the vertical dashed lines indicate the compositions at which electron percolations complete.

Table S15 │Electron mobilities of BTP-eC9 films diluted by PM6 in different ratios with the unit of 10^{-4} cm^2 V^{-1} s^{-1}.

BTP-eC9 content	100%	70%	40%	25%	20%	15%	10%
Control	9.87	6.41	4.65	1.52	0.20	0.016	0.0069
EEF-Coating	16.35	13.23	12.37	11.32	8.69	1.321	0.0408

Supplementary text S11:

When a voltage is applied to an organic heterojunction, electrons move between the materials. However, because electron conductivity differs between materials, electrons tend to move along the material with higher electron conductivity. This results in a percolation path for electrons in the heterojunction. The percolation threshold is a key concept that represents the conditions under which electrons begin to form percolation paths in the heterojunction. Specifically, the permeation threshold changes when the weight fractions of electron acceptors and electron donors change. Usually, the threshold of electron penetration is related to the following factors. The electron conduction properties of the material: If the electron conduction properties between the electron acceptor and the electron donor are very different, the percolation threshold may be lower. In other words, even with only a small number of electron acceptors, an effective electron percolation path can be formed. Interface and Doping: Interface properties in heterojunctions as well as the doping level of the material also affect the percolation threshold. Careful design and control of these factors can modulate the percolation threshold. The conjugated structure and conjugation length of electron acceptors have an important influence on the electron percolation threshold in organic heterojunctions. Conjugated structures usually involve conjugated bonds or π electron

clouds in organic molecules, and these structural elements play a key role in electron conduction properties. Conjugated structures often enhance the electronic conduction properties of organic molecules. This is because conjugated bonds or π electron clouds can easily support the movement of electrons, thereby increasing the mobility of electrons in the molecule. Therefore, electron acceptors with more conjugated structures usually have better electron conduction properties. This means that when the conjugated structure in the electron acceptor increases, the electron conduction performance may be higher, thereby influencing the electron percolation threshold. To obtain the electron percolation threshold, we measured the electron mobility at different NFA fractions in heterojunctions and fitted it with:

$$\mu = \mu_0 \exp\left\{-\frac{2D}{\xi}\left[\frac{0.15(a\,/\,b)}{\chi\phi}\right]^{4/3}\right\}$$

where D is the center-to-center distance between two conducting units. Here χ is the fraction of the acceptor moieties that can contribute to electron transport. χ values ranging from 0.3-0.4, 0.4-0.5, and 0.6-0.8 for fullerene, NFA, and polymer, respectively, suggest that all-polymer heterojunctions are favorable for transporting electrons. First, in heterojunctions with different acceptor contents, the electron mobility decreases as the mass fraction of the acceptor becomes smaller. When the electron acceptor mass fraction falls below the percolation threshold, a sudden decrease in electron mobility occurs by an order of magnitude. Based on this, we performed two linear fittings on the mobility in the active layer with different NFA mass fractions. The results showed that the electron mobility of the heterojunction in the control group dropped sharply when the acceptor ratio was reduced to 28%. For EEF-coated heterojunctions, the same

phenomenon only occurs when the acceptor ratio is reduced to 20%. This is because the electric field-induced NFA fibers strengthen the long-range ordered connections between small molecules in the mixture, thereby improving the electron mobility at high donor:acceptor ratios. Additionally, due to the electric field-induced formation of NFA fiber crystals in heterojunctions, the electron percolation threshold in small molecule acceptor-based heterojunctions reaches values comparable to those of polymer acceptor-based heterojunctions (**Fig. S42**). This means that in the blend phase with low acceptor fraction, the electrons can still rely on the long-range ordered NFA fiber crystals to maintain satisfactory mobility, thus effectively suppressing the carrier recombination loss.

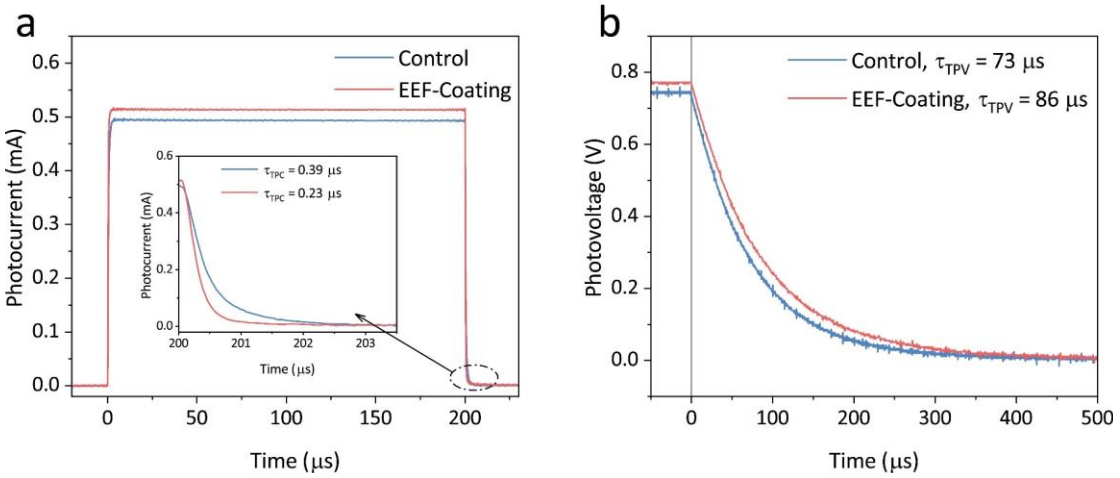

Figure S43 │ Electrical characteristics of charge carriers. a, Transient photocurrent of the corresponding devices. **b**, Transient photovoltage of the corresponding devices

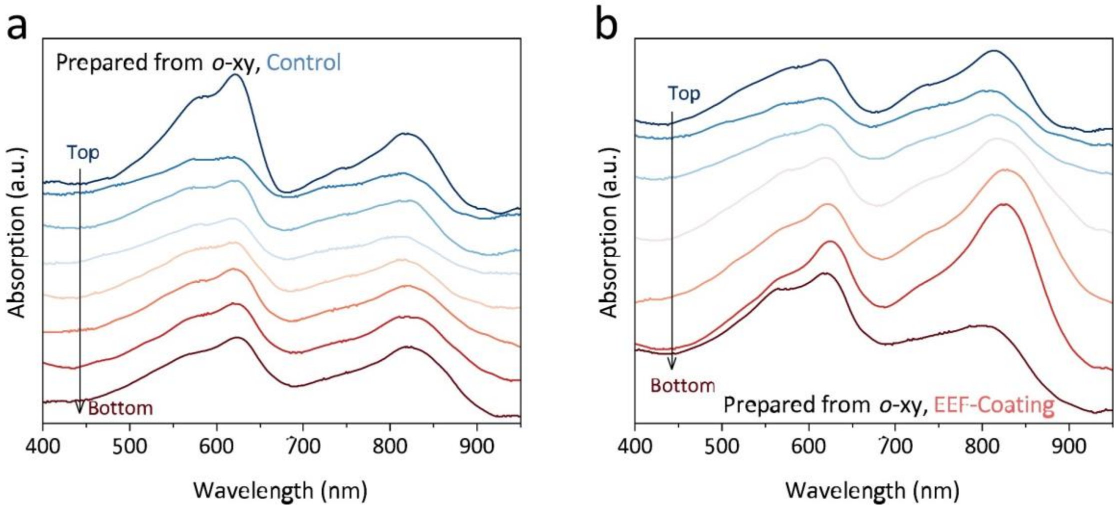

Figure S44 │ Film depth dependent optical investigations. **a**, Control and **b**, EEF-coated film-depth-dependent absorption spectrum. The bottom is the near-substrate side of the active layer, and top is the far-substrate side.

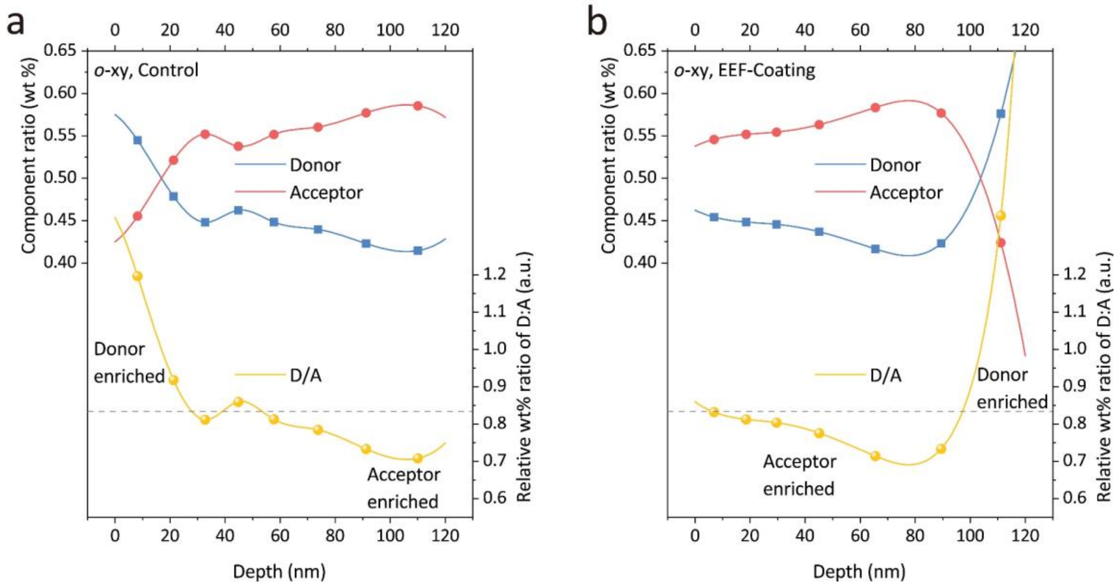

Figure S45 │ Film depth dependent optical investigations. **a**, Control and **b**, EEF-coated film-depth-dependent vertical gradient of components extracted from the *o*-xy processed sub-layers absorption spectrum. The depth of 120nm is the near-substrate side of the active layer, and 0nm is the far-substrate side.

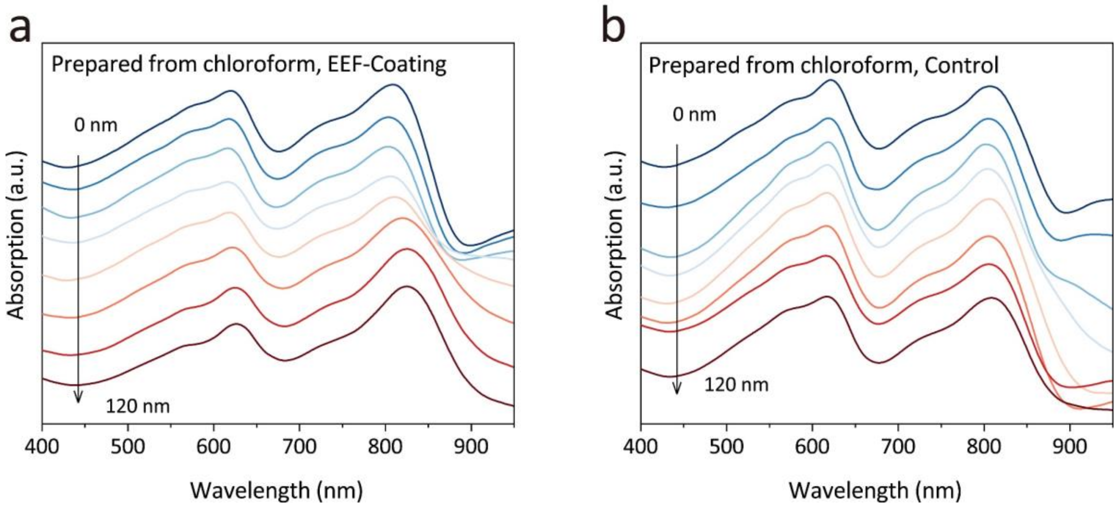

Figure S46 │ Film depth dependent optical investigations. a, Control and **b**, EEF-coated film-depth-dependent absorption spectrum. The bottom is the near-substrate side of the active layer, and top is the far-substrate side.

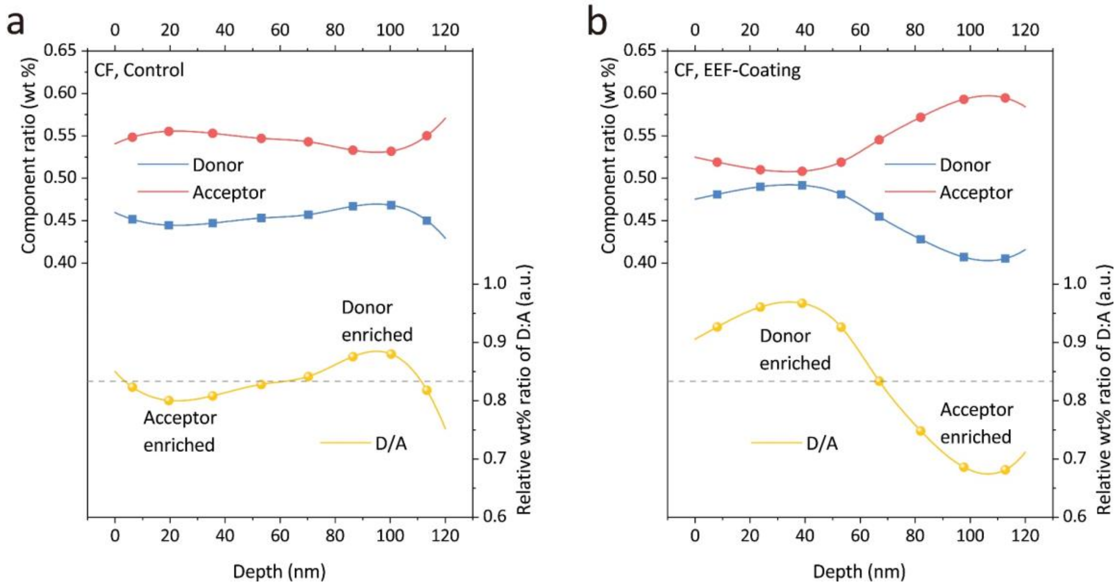

Figure S47 │ Film depth dependent optical investigations. a, Control and **b**, EEF-coated film-depth-dependent vertical gradient of components extracted from the CF processed sub-layers absorption spectrum. The depth of 120nm is the near-substrate side of the active layer, and 0nm is the far-substrate side.

Supplementary text S12:

The vertical distribution gradient of donor and acceptor materials within active layers have been demonstrated to be crucial factors in determining the photovoltaic

performances of OSCs. The film-depth-dependent absorptions reveal film-depth-dependent composition distributions (**Fig. S46**). When chloroform was used as the solvent, the electric field caused the surface layer to become a donor-enriched region, while the bottom layer become an acceptor-enriched region (**Fig. S47**). This vertical component redistribution contributes to the absorption redshift as we obtained above. This pattern of component distribution also explains why the application of EEF-coating to CF-processed inverted devices results in extremely significant photovoltaic performance. Combined with the role of EEF on the vertical component distribution pattern of the *o*-xy processed active layer, it can be concluded that *o*-xy as a solvent in the EEF-coating process is better suited with conventional devices, and CF is more suitable for inverted device structures.

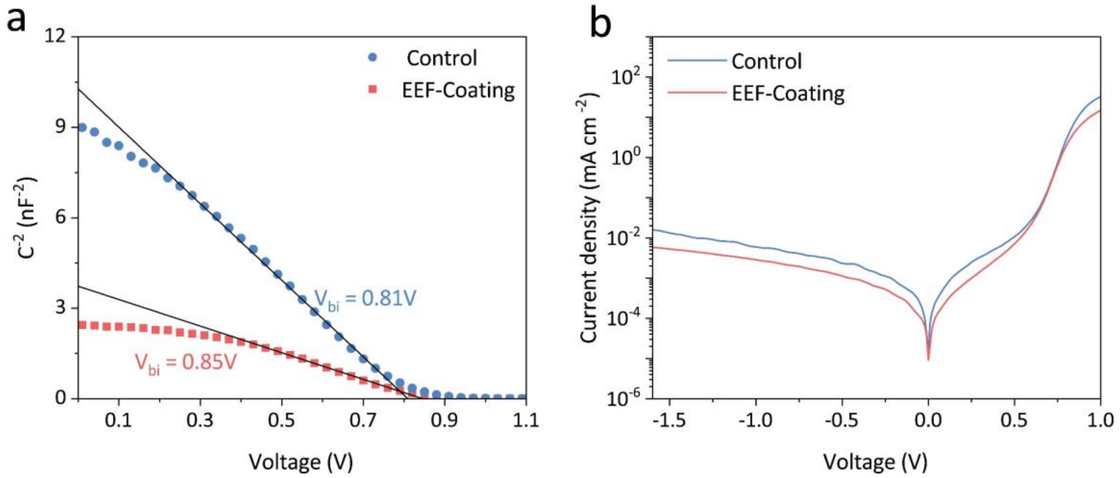

Figure S48 │ Electrical characteristics of OSCs. a, Mott-Schottky characteristics of the devices measured at 10 kHz in dark to determine the built-in potential. **b**, Current density-voltage characteristic curve measured in the dark.

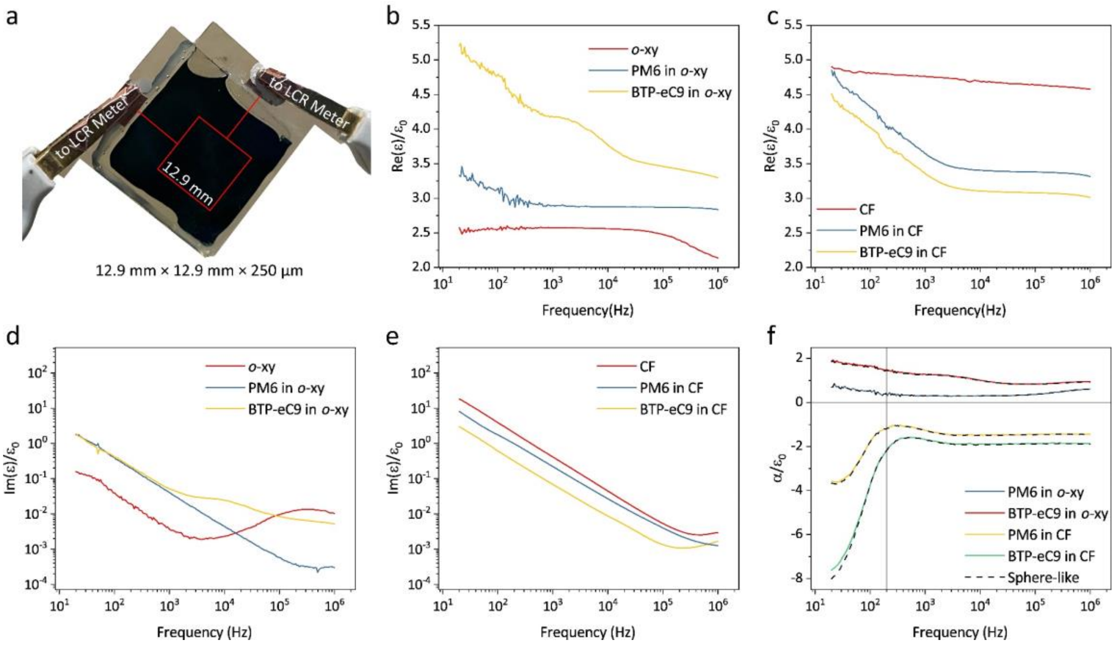

Figure S49 │ Dielectric spectroscopy of PM6, BTP-eC9, and solvents. a, Picture and electrical model of the apparatus based on a parallel-plate capacitor, utilized to perform dielectric spectroscopy of organic solvents and solutions (see Supplementary text S13). **b**,**c**, The real part of the dielectric of two common solvents (*o*-xylene and chloroform) and PM6 and BTP-eC9 solutions dissolved in each solvent measured at different frequencies. **d**,**e**, The imaginary part of the dielectric of two common solvents (*o*-xylene and chloroform) and PM6 and BTP-eC9 solutions dissolved in each solvent measured at different frequencies. **f**, Estimation of the polarizability for every working frequency and two solvents with different polarity, indicating their influence in DEP.

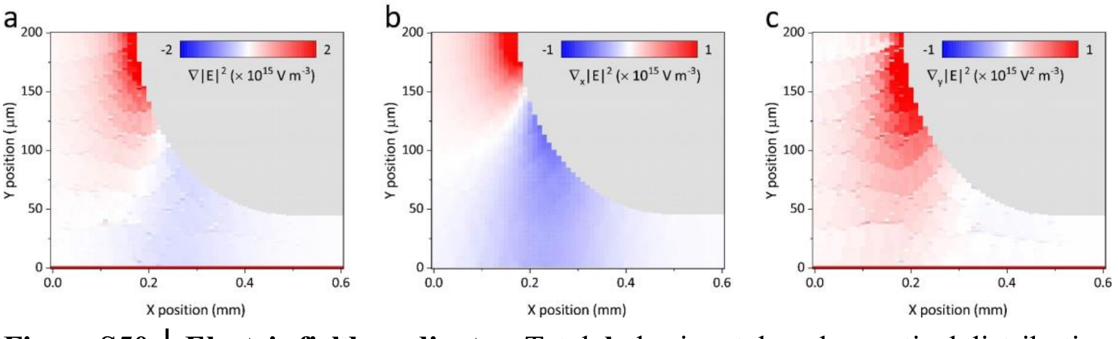

Figure S50 │ Electric field gradient. a, Total, **b**, horizontal, and **c**, vertical distribution of the calculated square of the electric field gradient in the meniscus during the blade coating process.

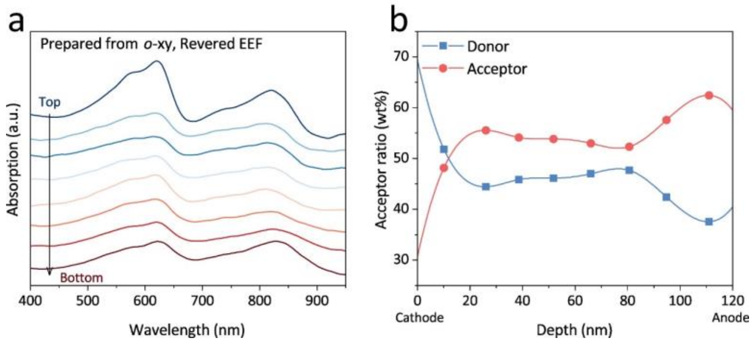

Figure S51 │ Film depth dependent optical investigations. a, **b**, Film-depth-dependent vertical gradient of components extracted from the *o*-xy processed sub-layers absorption spectrum. The depth of 120nm is the near-substrate side of the active layer, and 0nm is the far-substrate side. During the EEF-coating process, the high potential is set on the doctor blade and the zero potential is set on base plate.

Supplementary text S13:

Dielectric (organic photovoltaic material) in a non-uniform electric field, due to the different field strengths of the positive and negative electric centers, causes the particles to be stressed as a whole, that is, dielectrophoresis(*33*, *34*). Dielectrophoresis is a phenomenon in which a dielectric is subjected to a force in a non-uniform electric field. The existence of this force does not require the object itself to be electrically charged. All particles have dielectrophoresis in an electric field environment, but the magnitude of the force depends largely on the electrical properties of matter and particles, the shape and size of particles, and the rate of change of field intensity. Therefore, electric fields of specific frequencies can selectively control specific particles. It is applied in the separation of cells and in the orientation and control of nanoparticles and nanowires.

The time-averaged dielectrophoretic (DEP) force is described by the following formula:

$$\vec{F}_{DEP} = \frac{1}{2}\alpha v \nabla \vec{E}_{RMS}^{2}$$

where α is the polarizability, for the medium particles immersed in the solvent, it can be expressed as:

$$\alpha = Re(\varepsilon_s)Re(f_{CM})$$

Where f_{CM} is the Clausius-Mossotti factor:

$$f_{CM} = \frac{\varepsilon_p - \varepsilon_s}{\varepsilon_s + (\varepsilon_p - \varepsilon_s)L_i}$$

with

$$\varepsilon(\omega) = \varepsilon' - j\varepsilon''$$

L_i (i=x,y,z) is the depolarization factor related to particle shape. For spherical dielectric particles, the polarizability can be expressed as:

$$\alpha_{sph} = 3Re(\varepsilon_s) \cdot Re\left(\frac{\varepsilon_p - \varepsilon_s}{\varepsilon_p + 2\varepsilon_s}\right)$$

For ellipsoidal or rod-like dielectric particles with a long axis, the polarizability can be expressed as:

$$\alpha_{rod} = Re(\varepsilon_s) \cdot Re\left(\frac{\varepsilon_p - \varepsilon_s}{\varepsilon_s}\right)$$

The force direction is related to the positive or negative value of α/ε_0, in fact, it is related to the difference ε_p-ε_s of the complex permittivity of the particle and the solvent(*22*). The magnitude of the force is related to the value of the complex permittivity of the material. The direction of the DEP force depends on the positive or negative value of α. Positive α leads to a positive DEP force, in which the particles are pulled to the high electric field area, and vice versa, they are pushed to the low electric field area (**Fig. S50**). According to the estimation of the electric field strength used in this work and the drift speed of organic molecules under the gradient, the speed is about tens of nm s^{-1}.

Fig. S49a shows a photograph and electrical model of the device based on a parallel

plate capacitor with a hollow gap (200 μm) for performing dielectric spectroscopy of *o*-xy solvent and PM6, BTP-eC9 solutions. **Fig. S49b** and **S49c** depicts the dielectric constants measured for two solvents and the PM6, BTP-eC9 solution dissolved in solvent at different frequencies, with the real part on the **b** and the imaginary part on the **c**. The real part of the dielectric constant was obtained from the capacitance C values of the solvents and solutions using the standard equation for parallel-plate capacitors $Re(\varepsilon) = tC/(A\varepsilon_0)$, where A is the surface area of the capacitor electrodes (12.9 × 12.9 mm^2), t is the gap between the electrodes and ε_0 the vacuum dielectric constant; while the imaginary part of the dielectric constant was obtained by measuring the parallel resistance R_p, i.e. $Im(\varepsilon) = t/(\omega R_p A\varepsilon_0)$, where ω is the applied angular frequency. To extract the net contribution of the PM6, BTP-eC9 molecule to the total dielectric constant of the solution, dielectric mixture model was used. For a mixture of two dielectric materials, one of which is the solvent (*o*-xy) and the other is the inclusions in the matrix (PM6, BTP-eC9), the dielectric constant of the mixed solution can be described as: $\varepsilon_s^\lambda = \phi\varepsilon_P^\lambda + (1-\phi)\varepsilon_s^\lambda$, where φ is the volume fraction of PM6, BTP-eC9 in solution and λ is an empirical factor which specifies while when λ = -1 represents the serial mixing rule.

The difference in polarity between chloroform and *o*-xylene is the main factor leading to these conclusions. The dielectric constant (or polarity) of organic molecules (PM6 and BTP-eC9) is higher than that of *o*-xylene but lower than that of chloroform. It results in non-uniform pulsed electric fields able to exert DEP forces in different directions on organic molecules dissolved in the two solvents. The force direction is

related to the positive or negative value of $\alpha/\varepsilon 0$, in fact, it is related to the difference ε_p-ε_s of the complex permittivity of the particle and the solvent. Positive α leads to a positive DEP force, in which the particles are pulled to the high electric field area, and vice versa, they are pushed to the low electric field area. In o-xy (or CF), PM6 and BTP-eC9 exhibit positive (or negative) polarizability values. Consequently, when dissolved in o-xy (or CF), the non-uniform electric field exerts a DEP force that pulls (pushes) PM6 and BTP-eC9 molecules towards (away from) regions with high electric fields at nanoscale speeds. Notably, the force acting on BTP-eC9 is significantly stronger than that on PM6, resulting in acceptor molecules drift toward the upper (lower) portion of the meniscus as depicted in **Fig. S50** and deposited on the surface (bottom) of the solid film.

As we discussed, the pulsed external electric field serves two purposes, one is to apply torque to the dipoles of the organic molecules for orientation modulation (which is dependent on the direction of the electric field), and the other is to provide DEP force to modulate the vertical distribution pattern of the components (which is primarily dependent on the shape of the electric field and the polarity of the solvent). When the above conclusion is established, changes in the direction of the electric field will not affect the results of the vertical component distribution (**Fig. S51**). The shape of an electric field, as indicated by the density of electric field lines, is solely determined by the shapes of the positive and negative poles (such as the doctor blade and the plate). Therefore, changing the direction of the electric field does not alter the shape of the electric field, and consequently does not result in a change in DEP force or the vertical

distribution pattern of the components. Changes in the polarity of the solvent are the only circumstances under which the distribution pattern of the vertical component would alter.
